# A reductive uric acid degradation pathway in anaerobic bacteria

**DOI:** 10.1093/lifemeta/loaf031

**Published:** 2025-07-31

**Authors:** Zhi Li, Wei Meng, Zihan Gao, Wanli Peng, Zhandong Hu, Jianhao Zhang, Yining Wang, Xiaoxia Wu, Zipeng Zhao, Chuyuan Zhang, Zhuohao Tang, Zhujun Nie, Shaohua Wu, Benjuan Wu, Hui Zheng, Duqiang Luo, Yang Tong, Yiling Hu, Zehan Hu, Yifeng Wei, Yan Zhang

**Affiliations:** New Cornerstone Science Laboratory, School of Pharmaceutical Science and Technology, Tianjin University, Tianjin 300072, China; New Cornerstone Science Laboratory, School of Pharmaceutical Science and Technology, Tianjin University, Tianjin 300072, China; School of Life Sciences and Biotechnology, Shanghai Jiao Tong University, Shanghai 200240, China; School of Life Sciences and Biotechnology, Shanghai Jiao Tong University, Shanghai 200240, China; Tianjin First Central Hospital, Tianjin 300190, China; School of Life Sciences and Biotechnology, Shanghai Jiao Tong University, Shanghai 200240, China; New Cornerstone Science Laboratory, School of Pharmaceutical Science and Technology, Tianjin University, Tianjin 300072, China; New Cornerstone Science Laboratory, School of Pharmaceutical Science and Technology, Tianjin University, Tianjin 300072, China; New Cornerstone Science Laboratory, School of Pharmaceutical Science and Technology, Tianjin University, Tianjin 300072, China; New Cornerstone Science Laboratory, School of Pharmaceutical Science and Technology, Tianjin University, Tianjin 300072, China; School of Life Sciences and Biotechnology, Shanghai Jiao Tong University, Shanghai 200240, China; New Cornerstone Science Laboratory, School of Pharmaceutical Science and Technology, Tianjin University, Tianjin 300072, China; Tianjin First Central Hospital, Tianjin 300190, China; Tianjin First Central Hospital, Tianjin 300190, China; Department of Anesthesiology, National Cancer Center, Chinese Academy of Medical Sciences and Peking Union Medical College, Beijing 100021, China; College of Life Sciences, Institute of Life Science and Green Development and Hebei Innovation Center for Bioengineering and Biotechnology, Hebei University, Baoding, Hebei 071002, China; New Cornerstone Science Laboratory, School of Pharmaceutical Science and Technology, Tianjin University, Tianjin 300072, China; New Cornerstone Science Laboratory, School of Pharmaceutical Science and Technology, Tianjin University, Tianjin 300072, China; School of Life Sciences and Biotechnology, Shanghai Jiao Tong University, Shanghai 200240, China; Singapore Institute of Food and Biotechnology Innovation (SIFBI), Agency for Science, Technology and Research (A*STAR), Singapore 138669, Singapore; New Cornerstone Science Laboratory, School of Pharmaceutical Science and Technology, Tianjin University, Tianjin 300072, China; School of Life Sciences and Biotechnology, Shanghai Jiao Tong University, Shanghai 200240, China; Frontiers Science Center for Synthetic Biology (Ministry of Education), Tianjin University, Tianjin 300072, China; Key Laboratory of Systems Bioengineering (Ministry of Education), School of Chemical Engineering and Technology, Tianjin University, Tianjin 300072, China

**Keywords:** uric acid, yanthine, gout, CBT2.0

## Abstract

Uric acid (UA) is a key intermediate in purine degradation across diverse organisms, while its accumulation in humans leads to inflammation and gout disease. Aerobic organisms degrade UA via a well-known “oxidative pathway” involving dearomatization of the purine core catalyzed by UA oxidases or dehydrogenases. The ability to degrade UA is also widespread in anaerobic bacteria, including gut bacteria, although the mechanisms are incompletely understood. Here, we report the biochemical characterization of a recently identified UA degradation gene cluster from *Escherichia coli*, and show that it encodes a “reductive pathway” for UA degradation. In this pathway, UA is first reduced to 2,8-dioxopurine (yanthine) by a xanthine dehydrogenase homolog (XdhD), followed by dearomatization of the purine core catalyzed by a flavin-dependent reductase (YgfK). Stepwise cleavage of the pyrimidine and imidazole rings forms 2,3-diureidopropionate, and stepwise cleavage of the 2- and 3-ureido groups then forms 2,3-diaminopropionate, which is cleaved by a pyridoxal 5′-phosphate-dependent lyase (YgeX) to pyruvate and ammonia. The detection of yanthine in clinical serum samples from healthy individuals and significantly higher levels from gout patients suggests that yanthine is a physiologically relevant circulating metabolite. A probiotic *E. coli* Nissle strain was engineered for constitutive overexpression of the gene cluster, and oral administration in a uricase-knockout hyperuricemic mouse model significantly reduced the serum UA level and alleviated associated kidney injury, suggesting a potential route towards uricolytic probiotics.

## Introduction

**Figure 1:**
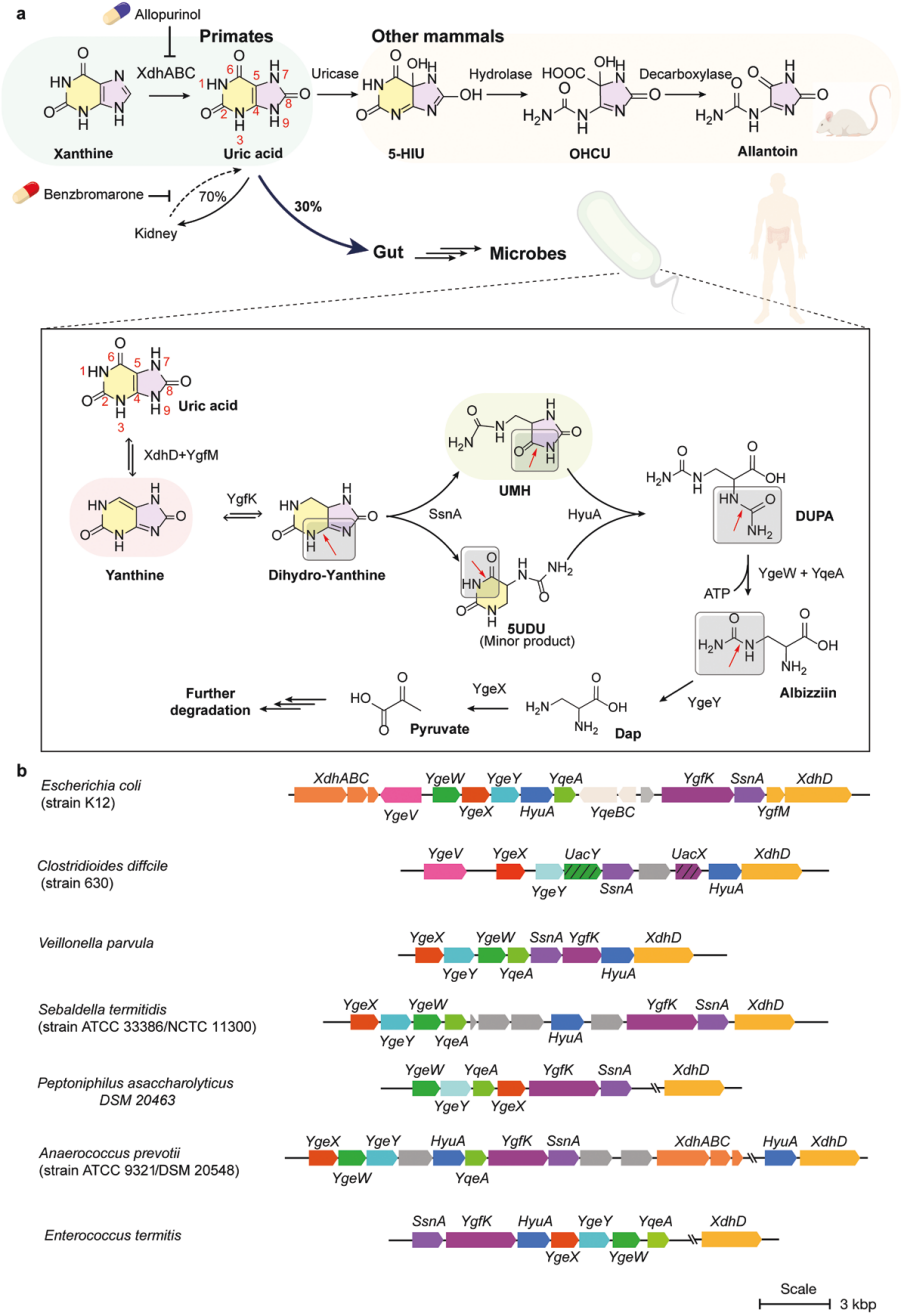
UA degradation pathways and gene clusters. (a) Upper panel, oxidative UA degradation pathway (previously known); lower panel, reductive UA degradation pathway (this work). The metabolic and excretory pathways of UA in mammals are depicted, with gout therapies and their molecular targets highlighted. (b) Representative reductive UA degradation gene clusters in bacteria. *E. coli* contains *XdhD* (PF01315, Ald_Xan_dh_C 1), *YgfK* (PF07992, Pyr_redox_2), *SsnA* (PF01979, Amidohydro_1), *HyuA* (PF01979, Amidohydro_1), *YgeW* (PF00185, OTCace), *YgeY* (PF01546, Peptidase_M20), and *YgeX* (PF00291, PALP). In *C. difficile*, all genes except *ygfK* and *ygeW* are fully conserved. In *C. difficile*, YgfK is replaced by *UacX* (PF01180, DHO_dh), and *YgeW* is replaced by *UacY* (PF07969, Amidohydro_3). *C. difficile XdhD* was assigned based on its proximity to uricolytic genes, despite low homology to *E. coli XdhD* (25.63%).

Uric acid (UA) is a ubiquitous intermediate in purine degradation, playing important roles in the physiology of various animals. In most mammals, the enzyme uricase converts UA into the more water-soluble allantoin, which is readily excreted [1, 2] (Fig. 1a). In contrast, humans and other higher primates lack a functional uricase and instead directly eliminate UA, primarily through urine, with a smaller contribution via the gut [3, 4] (Fig. 1a). In moderate levels, the electron-rich UA functions as a systemic antioxidant and supports neuroprotection [1, 5, 6]. However, elevated UA levels (hyperuricemia) can lead to a range of morbidities, and are treated pharmacologically with drugs like allopurinol or benzbromarone (Fig. 1a). Due to its poor solubility and high crystallinity, UA readily forms sodium urate crystals, which can deposit in the kidneys to cause kidney stones, or in joints to trigger gout, a form of inflammatory arthritis. Conversely, this low solubility is advantageous in reptiles, birds, and insects, where UA serves as the primary nitrogenous waste and is excreted in a semi-solid form, aiding in water conservation [7, 8].

The study of bacterial purine and pyrimidine degradation has a long and rich history, revealing diverse strategies for cleaving the aromatic N-heterocyclic cores. For pyrimidines, these include the oxidative and reductive pathways, where ring cleavage is preceded by uracil oxidative or reductive dearomatization [9, 10], as well as the alternative Rut (pyRimidine UTilization) [11, 12] and URC (URacil Catabolism) pathways [13, 14] (Supplementary Fig. S1a−d). The well-known purine degradation pathway generally involves deamination of adenine and guanine to hypoxanthine and xanthine, followed by oxidation to UA by the molybdenum cofactor (MoCo)-dependent enzyme xanthine dehydrogenase (XDH) [15, 16]. In aerobic organisms, UA is oxidatively dearomatized to 5-hydroxyisourate (5-HIU) by various urate oxidases and dehydrogenases [17–19], followed by hydrolysis and decarboxylation to form allantoin for further degradation [20] (Fig. 1a).

Purine and UA degradation pathways that operate independently of oxygen or external electron acceptors are of particular interest due to their relevance to the anaerobic human gut microbiome. The first such purine/UA degradation pathway was described in *Clostridia* [21] and elucidated by Rabinowitz *et al.* [22]. Here, XDH catalyzes the reversible reduction of UA to xanthine, which is then cleaved by xanthine amidohydrolase (xanthinase), followed by a series of hydrolytic and decarboxylative steps yielding glycine [23]. The gene cluster encoding this oxygen-independent “xanthinase pathway” was only recently identified, and found to occur in *Clostridia* and *Bacilli* [24] (Supplementary Fig. S1e). While mechanistically simple, its overall rate is constrained by the slow kinetics of xanthinase (*k*_*cat*_ = 0.1 s^−1^) [24]. UA is also degraded anaerobically by diverse bacteria from the UA-rich termite gut, despite lacking the xanthinase pathway [25–27]. *Streptococcus* UAD1 (now *Enterococcus termitis* UAD1) degrades UA when supplied with formate as a co-substrate, yielding CO_2_, NH_3_, and acetate [28]. Urea is not detected as an intermediate, and omission of formate leads to accumulation of a fluorescent metabolite in the culture medium.

The model bacterium *Escherichia coli* oxidizes purines to UA via a XDH homolog (XdhA), and is also capable of degrading allantoin [29]. However, it cannot oxidize UA to allantoin, thus lacking a complete pathway for aerobic purine degradation. Under anaerobic conditions, *E. coli* exhibits limited UA degradation in the presence of formate, previously proposed to involve two uncharacterized oxidoreductases (AegA [aerobic growth advantage protein A] and UacF [uric acid degradation formate-related element]) [30], located next to the uric acid-specific permease (UacT) [31]. Recent work by the laboratories of Liu *et al*. and Kasahara *et al*. provided a key advance by identifying a widely distributed gene cluster involved in anaerobic UA degradation, present in gut bacteria and contributing to host purine homeostasis [2, 32]. This gene cluster is adjacent to *XdhA* and *UacT* in the genome of *E. coli*, and is also present in other previously characterized anaerobic uricolytic bacteria [32, 33] (Fig. 1b), providing a basis for further investigations into the biochemical details of this pathway. In contrast to the uricase-dependent “oxidative pathway” (Supplementary Fig. S1f), we hypothesize that this gene cluster encodes a “reductive pathway” for UA degradation (Fig. 1a, boxed), analogous to the reductive pathway for pyrimidine degradation (Supplementary Fig. S1a).

## Results

### Comparative analysis of *E. coli* and *Clostridium difficile* uricolytic gene clusters

The *E. coli* uricolytic gene cluster encodes a XDH homolog (XdhD−YgfM), a flavoenzyme (YgfK), an amidohydrolase related to PydB (SsnA), D-stereospecific “phenylhydantoinase” (HyuA), a carbamoyltransferase (YgeW), a carbamate kinase (YqeA), a peptidase (YgeY), and a diaminopropionate ammonia lyase (YgeX), which is equally active on the L- and D-isomers [34]. These enzymes suggest a pathway in which the central C3 moiety of UA is released as diaminopropionate, requiring a net four-electron reduction and multiple hydrolytic steps (Fig. 1a, boxed).

Further insight was gained by comparing the *E. coli* gene cluster to that of *C. difficile*, which is also capable of degrading UA [33]. In *C. difficile*, YgfK is replaced by a homolog of PydA (which we designate UacX), an enzyme catalyzing reductive dearomatization of uracil in the pyrimidine degradation (Pyd) pathway (Fig. 1b; Supplementary Fig. S1a). Additionally, in *C. difficile*, YgeW and YqeA are replaced by a homolog of N-acyl-D-amino acid hydrolase (D-aminoacylase), which we designate UacY (Fig. 1b). Structural modeling reveals that its active site closely resembles that of the characterized D-aminoacylase from *Bordetella* (PDB: 3GIQ) with conserved residues binding the D-aminoacyl moiety (Supplementary Fig. S2a), suggesting that YgeW and UacY catalyze the cleavage of a similar N-acyl amino acid substrate.

Structural modeling suggests that *E. coli* XdhD−YgfM form a complex resembling *Rhodobacter capsulatus* XDH (PDB: 1JRO), with XdhD containing the MoCo-binding hydroxylase/dehydroxylase site, and YgfM containing the flavin adenine dinucleotide (FAD)-binding site for electron transfer from NAD(P)H (Supplementary Fig. S2b). Structural modeling also suggests that *E. coli* YgfK contains a flavin mononucleotide (FMN)-binding site likely for substrate reduction, and a FAD site likely for electron transfer from NAD(P)H, resembling the architecture of *Sus scrofa* PydA (Supplementary Fig. S2c). By contrast, *C. difficile* XdhD lacks the YgfM subunit, and *C. difficile* UacX contains an FMN-binding site but lacks the FAD-binding domain. Instead, the architecture of *C. difficile* UacX resembles Clostridial PydA (PydAc) [35], suggesting an alternative electron donor for both Clostridial enzymes, XdhD and UacX, such as reduced ferredoxin (Fdx).

Variants of the gene cluster are also present in other organisms previously reported for anaerobic UA degradation (Fig. 1b), including *Enterococcus faecalis* isolated from chicken cecum or human feces, *Peptoniphilus asaccharolyticus* DSM 20463, the RNA-degrading *Peptococcus prevotii* (now *Anaerococcus prevotii*) from human skin, and *Veillonella parvula* from human oral cavity.

Together, the composition of the two gene clusters offers key insights into the key steps involved in the proposed pathway (Fig. 1a, boxed). The net four-electron reduction likely involves a dehydroxylation step catalyzed by XdhD, and a reductive dearomatization step catalyzed by YgfK/UacX. Following dearomatization, the two rings are likely cleaved by SsnA and HyuA. The two ureido groups linked to the diaminopropionate moiety are likely cleaved by YgeY and YgeW/UacY, with YgeY cleaving the 3-ureido group, and YgeW/UacY cleaving the 2-ureido group, as suggested by the substrate specificity of D-aminoacylases.

### XdhD catalyzes the reduction of UA to 2,8-dioxopurine (yanthine)

Potrikus and Breznak reported that in the absence of formate as a co-substrate, *Enterococcus* UAD1 degrades UA incompletely, secreting a fluorescent compound with excitation and emission maxima at 308 and 379 nm (pH 7.0), respectively [36]. To identify potential intermediates in the pathway, we screened a panel of aromatic metabolites, including UA, hypoxanthine, xanthine, yanthine (2,8-dioxopurine), 2,6-dioxopurine, 5-aminouracil, and 5-ureidouracil, and found that only yanthine displayed a closely matching fluorescence emission peak at 363 nm upon excitation at 308 nm (Supplementary Fig. S3a and b). We therefore hypothesized that yanthine is an intermediate, formed via dehydroxylation of UA catalyzed by XdhD.

**Figure 2:**
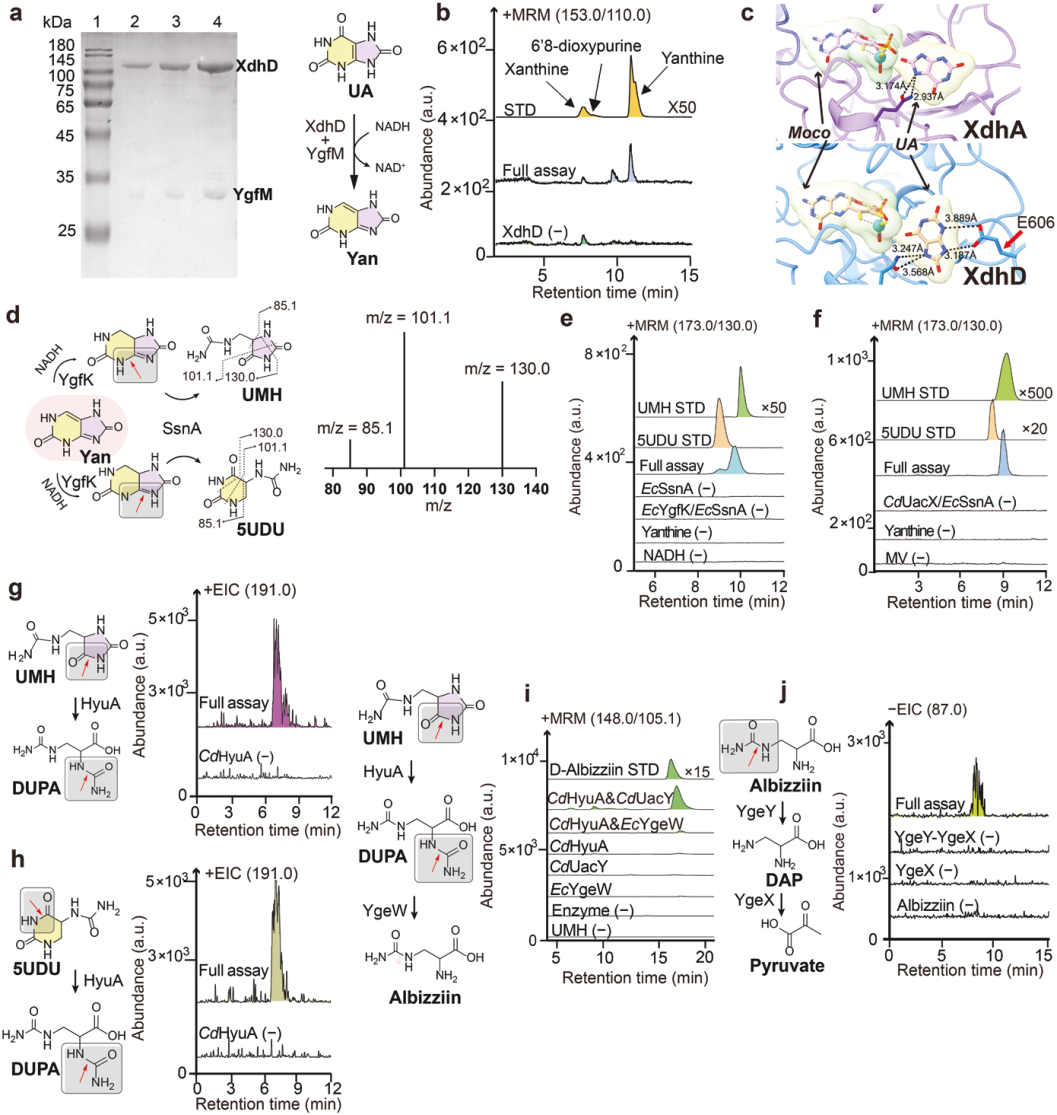
Characterization of enzymes in the reductive UA degradation pathway. (a) SDS-PAGE of moderately overexpressed *Ec*XdhD, showing copurification with an approximately 30 kDa protein, putatively YgfM. Lane 1: molecular weight marker; lanes 2−4: 1, 2, 4 μg protein loaded, respectively. (b) Assays of *Ec*XdhD−YgfM, showing reduction of UA to yanthine. (c) Active site models of *Ec*XdhA and *Ec*XdhD, highlighting the potential role of *Ec*XdhD Glu606 in substrate orientation. (d−f) Coupled assay of either *Ec*YgfK or *Cd*UacX with *Ec*SsnA, showing the conversion of yanthine to UMH (major product) and 5UDU (minor product). (g and h) Assays of *Cd*HyuA, showing hydrolysis of both UMH and 5UDU to DUPA. (i) Coupled assays of *Cd*HyuA with either *Cd*UacY or *Ec*YgeW, showing the conversion of UMH to albizziin. (j) Coupled assays of recombinant *Ec*YgeY and *Ec*YgeX, showing the conversion of albizziin to pyruvate. Chromatogram traces were vertically offset, with standard intensities scaled down by the indicated factors to aid comparison.

To test this hypothesis, *E. coli* XdhD was expressed with a C-terminal ProtA affinity tag from the low-copy plasmid *p*SC101, to prevent overwhelming the cofactor biosynthetic pathway. Affinity purification of XdhD showed co-purification with a protein of approximately 30 kDa, putatively YgfM (Fig. 2a). Incubation of the purified protein with UA and NADH resulted in the formation of yanthine as the major product, as identified by liquid chromatography-mass spectrometry (LC-MS) and co-elution with a commercial standard (Fig. 2b). Boltz-1 prediction of XdhD in complex with UA and structural comparison reveal that E606 which is present in the active site of XdhD but absent in XdhA may contribute to the orientation of the substrate and formation of yanthine (Fig. 2c).

### YgfK and SsnA catalyze reductive dearomatization and ring cleavage of yanthine

The remaining enzymes in the pathway were individually overexpressed and purified (Supplementary Fig. S4). The YgfK 4Fe-4S clusters were reconstituted anaerobically and characterized by ultraviolet-visible (UV-Vis) absorption spectra [37, 38] (Supplementary Fig. S5a). Activity assays of individual enzymes with yanthine showed no detectable activity. However, anaerobic incubation of yanthine with YgfK, SsnA, and NAD(P)H resulted in the formation of ureidomethyl-hydantoin (UMH) as the major product and 5-ureido-dihydrouracil (5UDU) as a minor product, as identified by LC-MS and co-elution with commercial standards (Fig. 2d–f). These results are consistent with reductive dearomatization of yanthine by YgfK, followed by hydrolytic cleavage by SsnA. Failure to detect the reduction product with YgfK alone may reflect the unfavorable thermodynamics of this reaction, which requires coupling to hydrolysis by SsnA, consistent with the co-localization of the two genes in many organisms (Fig. 1b). A possible catalytic mechanism for formation of the two SsnA products is shown in Supplementary Fig. S5b.

### Enzymes catalyzing the cleavage of UMH to diaminopropionate

Given the structural similarity of UMH and 5UDU to known HyuA substrates, we assayed HyuA activity using the commercially available racemic compounds, and confirmed cleavage of both by LC-MS (Fig. 2g and h). Incubation of UMH with HyuA, in the presence of either UacY or YgeW and phosphate, resulted in the formation of 3-ureido-2-aminopropionate (albizziin), as confirmed by LC-MS and co-elution with a commercial standard (Fig. 2i). The reaction peak with YgeW was notably smaller than that with *Cd*UacY, likely reflecting the less favorable thermodynamics of the phosphorylysis reaction. Finally, incubation of albizziin with YgeY and YgeX yielded pyruvate as the terminal product of the pathway (Fig. 2j).

### Variants of the uricolytic gene cluster in different bacteria

**Figure 3:**
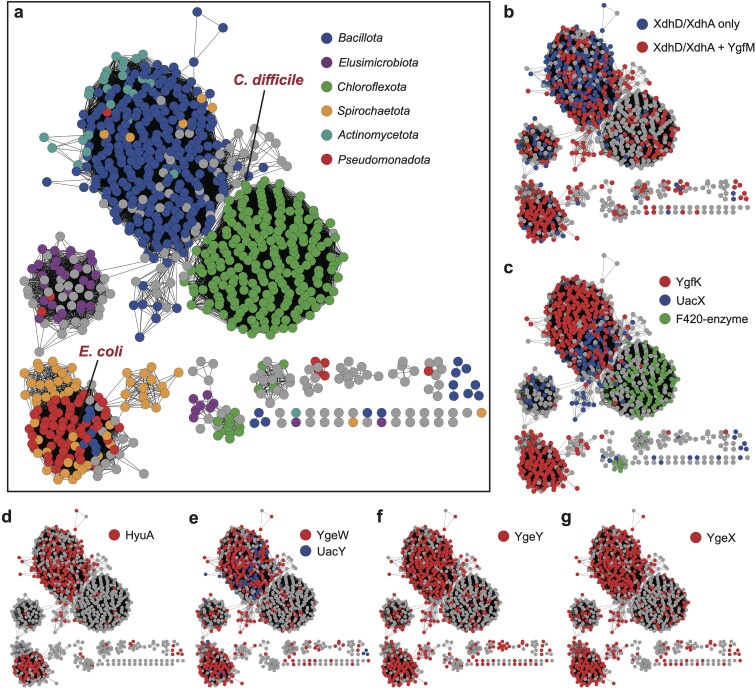
SSN of the SsnA family (IPR017700) in bacteria. (a) Nodes colored according to bacterial phyla: blue (*Bacillota*), red (*Pseudomonadota*), green (*Chloroflexota*), purple (*Elusimicrobiota*), orange (*Spirochaetota*), teal (*Actinomycetota*). (b−g) Nodes colored according to the presence of specific protein families of the different uricolytic enzymes within a 15-gene neighborhood. (b) XdhD or XdhA (PF01315) with or without YgfM (PF00941). (c) YgfK (IPR017701), UacX (PF01180), or putative F420-dependent reductase (PF00296). (d) HyuA (IPR011778). (e) YgeW (PF00185) or UacY (PF07969). (f) YgeY (PF01546). (g) YgeX (PF00291).

SsnA is one of the most identifiable uricolytic enzymes, catalyzing a key step in the pathway and serving as a robust marker for identifying uricolytic gene clusters. To explore the distribution of uricolytic gene clusters across bacteria, we constructed a Sequence Similarity Network (SSN) of the SsnA family (IPR017700). SsnA is prevalent in *Pseudomonadota* (e.g. *E. coli*), *Bacillota* (e.g. *C. difficile*), as well as *Chloroflexota* and *Actinomycetota*. It is also present in *Spirochaetota* and *Elusimicrobiota*, two phyla commonly found in the termite gut (Fig. 3a). Genome neighborhood analysis within a 15-gene window revealed that SsnA colocalizes with the upstream pathway genes. These include XdhA/XdhD homologs, some containing the YgfM FAD-binding domain (Fig. 3b), and either YgfK (as in *E. coli*) or UacX (as in *C. difficile*) (Fig. 3c). Notably, *Chloroflexota* lack these enzymes and instead contain a putative F420-dependent dehydrogenase belonging to the “luciferase family”. suggesting an alternative mechanism for yanthine reduction (Fig. 3c). In many organisms, SsnA also colocalizes with the downstream pathway genes *HyuA* (Fig. 3d), *YgeW/UacY* (Fig. 3e), *YgeY* (Fig. 3f), and *YgeX* (Fig. 3g). These findings suggest the widespreadness of the uricolytic pathway in different bacterial lineages.

### An engineered *E. coli* strain CBT2.0 overexpressing the uricolytic gene cluster

Recent studies by Liu *et al*. demonstrated that microbiota depletion in uricase-deficient mice leads to hyperuricemia, and that treatment with antibiotics targeting anaerobic microbiota increases the risk of gout in humans [2]. Kasahara *et al*. further showed that colonizing gnotobiotic mice with purine-degrading bacteria modulates systemic levels of UA, and that uricolytic bacteria harboring this gene cluster are correlated with serum UA levels in humans [32]. By extension, we hypothesized that engineering gut bacteria for constitutive overexpression of the genes in the uricolytic pathway could enhance uricolytic activity by the gut microbiome, and ensure consistent pathway activation while bypassing repression by energy-rich dietary molecules such as glucose.

**Figure 4:**
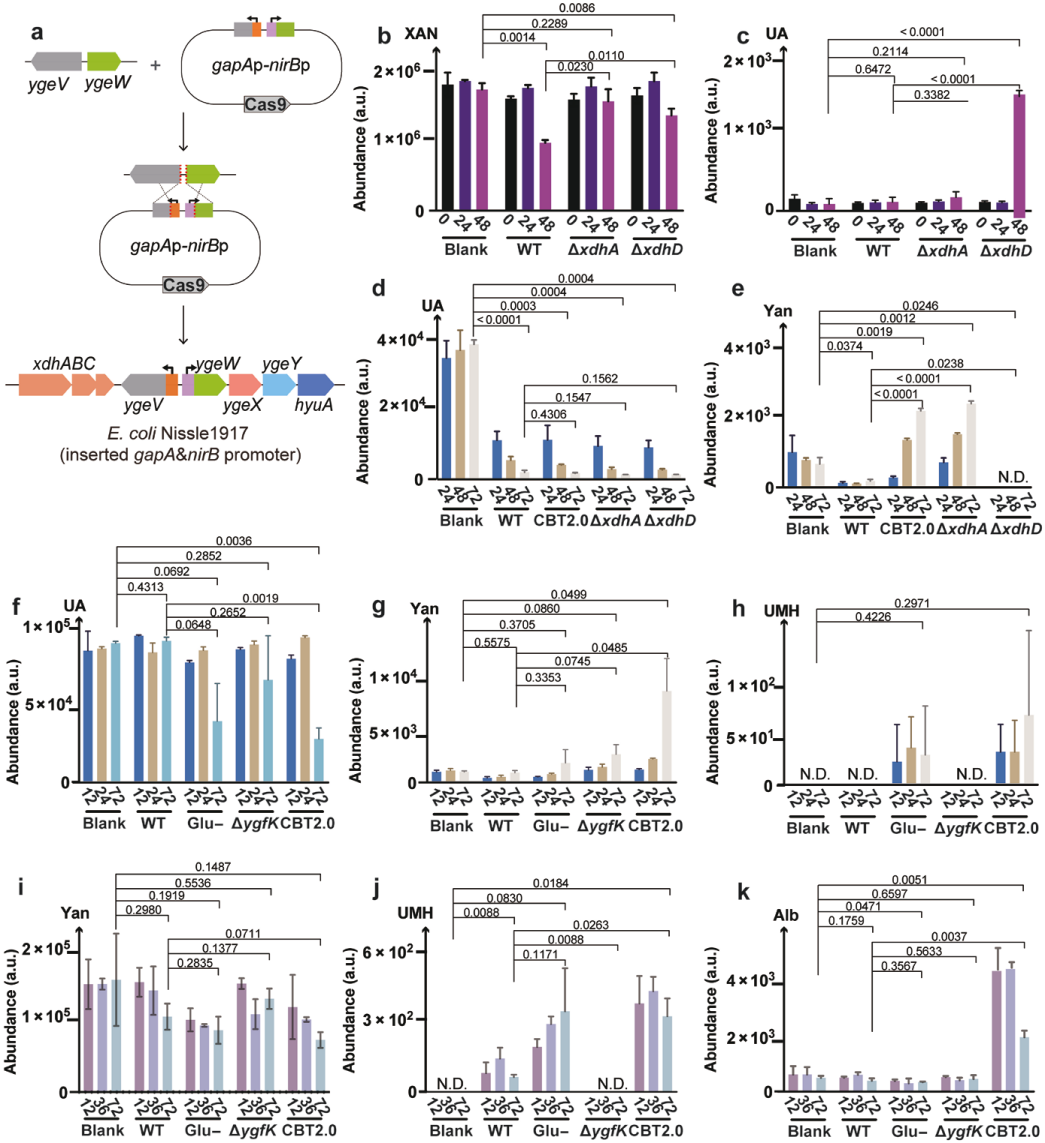
Detection of anaerobic UA degradation and resulting metabolites in *E. coli* mutant strains. (a) Construction of CBT2.0 with constitutive expression of the reductive UA degradation pathway. (b and c) Xanthine consumption and UA accumulation in various *E. coli* K12 MG1655 strains grown anaerobically in minimal medium. (d and e) UA consumption and yanthine accumulation in various *E. coli* Nissle 1917 strains grown anaerobically in LB medium. (f−h) UA consumption and metabolic intermediate accumulation in various *E. coli* Nissle 1917 strains grown anaerobically in LB media. (i−k) Yanthine consumption and metabolic intermediate accumulation in various *E. coli* Nissle 1917 strains grown anaerobically in LB medium.

Starting with the parental *E. coli* strain *EcNc*, derived from the probiotic *E. coli* Nissle 1917 (*EcN*) by ejection of two cryptic plasmids pMUT1 and pMUT2, CRISPR-Cas9 genome editing was used to insert a hybrid promoter cassette comprising (i) a strong constitutive *gapA* promoter and (ii) an anaerobic *nirB* promoter into the intergenic region between *ygeV* and *ygeW*, replacing the native Crp (cAMP receptor protein)-binding site (Fig. 4a). The resulting strain was named CarBT4gout 2.0 (CBT2.0, Fig. 4a), and overexpression of uricolytic genes was confirmed by RNA-seq analysis (Supplementary Fig. S6). For more detailed biochemical analysis, the Δ*xdhA* and Δ*xdhD* deletion mutants were generated for *E. coli* strains MG1655 (Fig. 4b and c) and CBT2.0 strains (Fig. 4d and e). Δ*ygfK* deletion mutants were also generated for *EcN* (Fig. 4f–k).

Liu *et al*. previously showed that gut bacteria metabolize UA via two distinct pathways: reduction to xanthine (via XdhA) or uricolysis (via SsnA, YgfK, etc.) [2]. To evaluate the proposed uricolytic pathway *in vivo*, we cultured selected mutant strains with the various purine compounds, collected culture supernatants at defined time points, and analyzed them by LC-MS. For cultures in M9 glucose medium supplemented with xanthine, both WT and Δ*xdhD* consumed xanthine, while Δ*xdhA* did not, consistent with the role of XdhA in xanthine oxidation (Fig. 4b). In Δ*xdhD*, xanthine consumption was accompanied by UA accumulation, consistent with the role of XdhD in subsequent UA degradation (Fig. 4c).

For cultures with Luria-Bertani (LB) medium supplemented with UA, all strains tested showed UA depletion, likely via reduction by XdhABC to xanthine, or reduction by XdhD−YgfM to yanthine, which was further degraded (Fig. 4d). XdhABC and XdhD−YgfM are not functionally redundant but act sequentially in purine degradation. Specifically, deletion of XdhD prevents UA degradation via the XdhD−YgfM pathway; instead, UA is redirected towards the purine salvage pathway mediated by XdhABC. Yanthine was detected at low levels in fresh LB medium, and further accumulated only in CBT2.0 and Δ*xdhA*, suggesting increased flux through XdhD in these strains (Fig. 4e). Consistently, no yanthine was observed in Δ*xdhD* (Fig. 4e). For cultures with LB supplemented with UA and glucose, UA degradation was repressed in WT but not in CBT2.0, indicating that CBT2.0 can bypass glucose repression (Fig. 4f). Yanthine accumulated in CBT2.0 (Fig. 4g), similar to the observations in LB without glucose (Fig. 4e). UMH was detected only in CBT2.0 and in WT grown without glucose, conditions where UA degradation occurred, supporting its role as a downstream intermediate (Fig. 4h). For cultures with LB supplemented with yanthine and glucose, all strains tested showed yanthine degradation, but degradation was faster in CBT2.0 and in WT grown without glucose (Fig. 4i), mirroring the trends with UA (Fig. 4f). UMH was detected for all strains except Δ*ygfK*, consistent with the role of YgfK in converting yanthine to UMH (Fig. 4h and j). Albizziin was detected at low levels in fresh LB medium, and further accumulated only in CBT2.0, possibly reflecting enhanced activity of upstream enzymes such as YgeW (Fig. 4k). Overall, the detection of yanthine, UMH, and albizziin supports their roles as intermediates in UA degradation, while the phenotypes of Δ*xdhA*, Δ*xdhD*, and Δ*ygfK* validate their proposed functions. Additionally, the CBT2.0 strain demonstrated resistance to glucose repression and increased flux through the uricolytic pathway under *in vitro* conditions in LB medium (Fig. 4f).

### *E. coli* CBT2.0 lowers UA levels in a hyperuricemic mouse model

**Figure 5:**
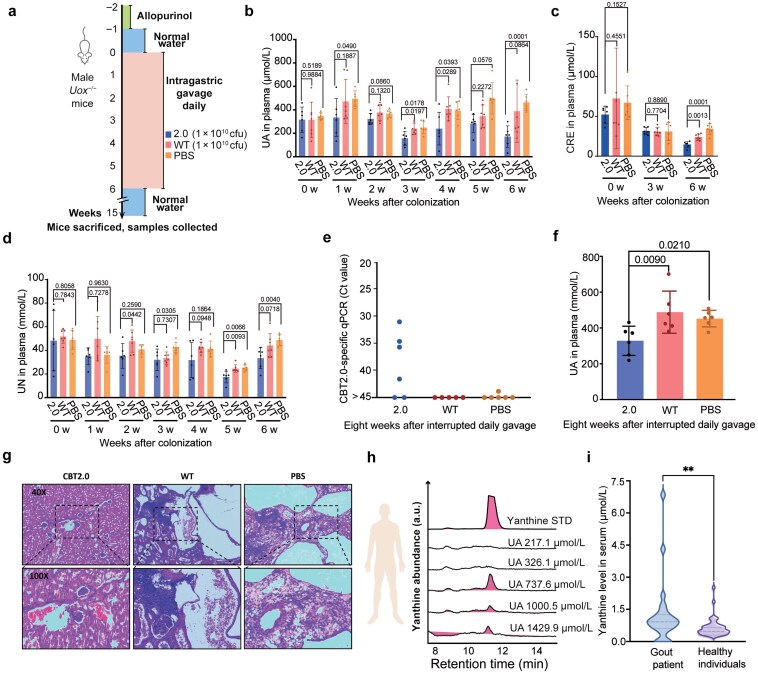
Evaluation of engineered probiotics in a murine gout model and detection of yanthine in human plasma. (a) Mouse experimental design. Male uricase-deficient (*Uox*^*−/−*^) mice were withdrawn from allopurinol at week −1 and then orally gavaged once daily for 6 weeks starting from week 0 with CBT2.0 (2.0, blue, 1 × 10^10^ CFU), wild-type *E. coli* Nissle 1917 (WT, red, 1 × 10^10^ CFU), or vehicle control (PBS, orange, 200 μL). Plasma samples were collected weekly. (b) Plasma UA levels in mice gavaged with 2.0, WT, or PBS. (c) Plasma CRE levels in mice gavaged with 2.0, WT, or PBS at week 6. (d) Plasma UN levels in mice gavaged with 2.0, WT, or PBS. The week-to-week variability in metabolite levels may be a result of different sampling times and circadian rhythms, and each weekly dataset was internally controlled. Bar graphs represent mean ± SD from *n* = 6−7 mice per group, with individual values overlaid. Statistical comparisons were performed with two-tailed unpaired *t*-tests, and exact *P* values are indicated above the brackets. See also Supplementary Table S6. (e) Detection of engineered CBT2.0 in mouse colonic contents by RT-qPCR. Y-axis represents Ct values obtained from primers targeting the specific gene of CBT2.0 strain. Each data point corresponds to an individual mouse (*n* = 5−6 per group) and represents the mean of three technical replicates. See also Supplementary Table S1. (f) Plasma UA levels in mice at week 15. (g) Representative H&E staining of kidney tissues from mice in CBT2.0, WT, and PBS groups. Upper pannels: 40 × magnification; lower panels: 100 × magnification. See also Supplementary Table S1. (h and i) Yanthine levels detected across human plasma samples with different UA levels. The clinical cutoff values for diagnosis of hyperuricemia are 416 μmol/L for males and 357 μmol/L for females, respectively. The week-to-week variability in mouse metabolite levels may be a result of different sampling times and circadian rhythms, and each weekly dataset was internally controlled.

To assess the UA-lowering potential of CBT2.0 in a host organism, we utilized a uricase-deficient (*Uox*^*−/−*^) mouse model of hyperuricemia. Mice were randomly assigned to three groups, and received daily intragastric gavage of CBT2.0, wild-type *EcN* (WT), or PBS vehicle control for 6 weeks, with weekly blood collection to monitor plasma levels of UA, urea nitrogen (UN), and creatinine (CRE) (Fig. 5a). Throughout the treatment period, mice administered CBT2.0 exhibited a significant reduction in plasma UA levels compared to the PBS control group. By week 6, the CBT2.0 group showed a mean UA concentration of 171.63 ± 91.59 μmol/L, whereas the PBS group maintained elevated levels at 463.26 ± 70.81 μmol/L (*P* < 0.001) (Fig. 5b). In contrast, the WT group showed a more modest reduction in UA levels compared to the PBS group at certain time points, but not consistently throughout the entire study period (Fig. 5b). These findings indicate that, compared to WT, CBT2.0 more reliably lowers systemic UA levels in *Uox*^*−/−*^ mice.

In urate oxidase-deficient mice, urate crystal deposition leads to earlier and more severe kidney damage than in humans, characterized by mechanical blockage of renal tubules and subsequent obstructive nephropathy [39]. Impaired renal excretory function typically results in systemic accumulation of CRE and UN. To evaluate the renal effects of the three treatments, we monitored plasma UN throughout the study and assessed CRE levels at weeks 0, 3, and 6. By week 6, the CBT2.0 group exhibited significantly lower CRE levels (14.61 ± 3.97 μmol/L) compared to the WT group (23.86 ± 3.81 μmol/L, *P* = 0.0013) and PBS group (34.14 ± 6.66 μmol/L, *P *< 0.0001) (Fig. 5c). A parallel trend was observed in UN levels, with the CBT2.0 group demonstrating markedly reduced level (33.40 ± 8.96 mmol/L) relative to the PBS group (48.75 ± 4.70 mmol/L, *P* = 0.004) and WT group (44.02 ± 10.07 mmol/L, *P* = 0.0718) (Fig. 5d). Notably, CBT2.0-treated mice displayed lower UA levels than the PBS group as early as week 1 (although statistical significance was not reached at weeks 2 and 5, *P* > 0.05) (Fig. 5b), and a significant UN reduction in the CBT2.0 group compared to PBS controls was first observed at week 3 (Fig. 5d), while no significant difference in CRE levels was detected at this time point (Fig. 5c), which suggest that the renal protective effects of the CBT2.0 treatment, potentially mediated through UA reduction, may exhibit a time-lagged progression. Subsequently, to determine the colonization of CBT2.0 and its long-term effects on *Uox*^*−/−*^ mice, we ceased daily gavage at week 6, and euthanized all mice at week 15 and collected their plasma, kidneys, and colonic contents, respectively. Despite a prolonged period (8 weeks) of interrupted probiotic treatment, the enrichment of a DNA fragment uniquely present in the CBT2.0 genome was still observed in the colon contents of four out of six mice in the CBT2.0 treatment group by real-time quantitative PCR (RT-qPCR) analysis, with a mean cycle threshold (Ct) value of 35.75 ± 4.38 (Fig. 5e). As expected, this sequence was not observable in the colon contents of the control groups (PBS and WT) with the Ct values higher than 45 (Fig. 5e; Supplementary Table S1). Control RT-qPCR assays targeting a fragment of 16S ribosome DNA (16S rDNA) that is conserved across bacterial species resulted in Ct values ranging from 10.70 to 16.03 for individual samples, with no significant variations among the three groups (Supplementary Fig. S7a; Supplementary Table S1). The ΔCt values indicating the relative abundance of CBT2.0 to all intestinal bacteria are also presented (Supplementary Fig. S7b). The plasma UA levels in the CBT2.0 group remained significantly lower than the WT group (*P *= 0.009) and PBS group (*P *= 0.021) (Fig. 5f). Within the CBT2.0 treatment group, the relative abundance of the CBT2.0-specific sequence to the 16S rDNA was negatively correlated with UA levels, which further highlighted the role of CBT2.0 in reducing UA in mice (Supplementary Table S1). The kidney morphologies were strikingly different among the three groups with the CBT2.0-treated mice being more normal-looking (Supplementary Fig. S8a). We next conducted histological analysis of these kidneys using hematoxylin&eosin (HE) staining. The results revealed that all mice exhibited varying degrees of renal cystic dilation and neutrophilic infiltration, with the degree of renal pathological changes in the CBT2.0 treatment group being significantly milder than the WT and PBS groups (Fig. 5g; Supplementary Fig. S8b; Supplementary Table S1). Collectively, oral treatment of CBT2.0 significantly reduced UA levels and alleviated UA-induced kidney injury, and colonization of this probiotic further exhibited long-term effects.

### Detection of yanthine in clinical serum samples

The detection of yanthine secretion in *E. coli* and previously in *Enterococcus* sp. [36] prompted us to investigate whether it is also detectable in serum and whether it correlates with UA levels. Using our in-house liquid chromatography-multiple reaction monitoring-mass spectrometry (LC-MRM-MS) assay, the newly discovered metabolite yanthine was sensitively quantified in the serum via the optimized transition *m/z* 153.0/110.0. A preliminary analysis of human serum samples with known UA levels suggested that yanthine levels could be used to distinguish three diagnosed gout patients from two healthy controls (Fig. 5h). In a subsequent analysis of 68 clinical human serum specimens (Fig. 5i), serum yanthine levels in 25 patients with gout were significantly elevated compared to 43 non-gout controls, with a statistically significant difference between the two groups (*P *< 0.01). This suggests that yanthine may serve as a biomarker, warranting further investigation.

## Discussion

Characterization of the enzymes encoded by a widely distributed anaerobic UA degradation gene cluster in *E. coli* reveals a “reductive pathway” for purine catabolism. All three purine degradation pathways studied to date initiate ring cleavage at the pyrimidine moiety, but employ different strategies to overcome the aromatic stabilization. In the “xanthinase pathway”, annelation to the aromatic imidazole ring diminishes the aromatic stabilization of the uracil moiety of xanthine, thereby permitting direct hydrolytic cleavage, albeit with slow kinetics [24]. Conversely, in the “reductive pathway”, the imidazole ring is dearomatized due to the 8-oxo group in yanthine, which facilitates subsequent reductive dearomatization of the uracil moiety and subsequent cleavage, in a manner directly analogous to PydA and PydB in the reductive Pyd pathway.

The molecular details of this pathway have important implications for understanding purine and UA metabolism in the anaerobic human gut microbiota, with potential clinical implications relevant to hyperuricemia and gout. While early studies focused on uricolytic bacteria from insects and birds, which excrete large amounts of UA, gut excretion is also a significant route of UA elimination in humans and other primates. Recent work by the laboratories of Dodd and Rey demonstrated that this gene cluster is broadly distributed among phylogenetically diverse human gut bacteria and can influence host purine and UA homeostasis [2, 32]. A notable observation was the detection of yanthine in human plasma samples. Yanthine was released by *E. coli* grown on UA, and likely corresponds to an unidentified fluorescent metabolite previously observed in *Enterococcus* sp. grown on UA in the absence of formate [36], showing that anaerobic bacterial purine metabolism can produce circulating metabolites, as previously reported for aromatic amino acid metabolism [40]. It is currently unclear whether yanthine has any biological activity. Given its strong fluorescence, yanthine merits further investigation as a potential biomarker for purine metabolic disorders, presumably more advantageous and convenient than enzyme-dependent UA measurement.

A widely proposed goal is to harness microbiome-based strategies to modulate UA cycling [41, 42], yet the regulation of UA metabolism genes and the metabolic flux of UA degradation across diverse gut bacteria remains poorly understood, particularly within the chemically complex environment of the digestive tract. Our experiments with an engineered strain of the probiotic *E. coli* Nissle in a uricase-deficient mouse gout model showed that constitutive expression of the UA degradation gene cluster led to reduced host plasma UA levels. It is known that in healthy human individuals, approximately 30% of UA is excreted through the intestinal tract [2]. This excretion route becomes more important and accounts for up to 2/3 of total UA elimination in patients with kidney malfunction [43]. We note that the *Uox*^−/−^ mouse model indeed exhibits inherent renal dysfunction from birth [39]. Therefore, a higher beneficial effect of CBT2.0 in plasma UA reduction is perhaps not so surprising. UA sequestered and degraded by CBT2.0 reduces the UA level in the intestinal lumen. Consequently, this may stimulate the transport of UA via the ATP-binding cassette subfamily G member 2 (ABCG2), increasing the flux of UA to the intestinal tract. Reduction of UA by CBT2.0 may also negatively regulate UA reabsorption, although the transporter has not been identified. Furthermore, UA elimination through CBT2.0 results in lower UA levels in the blood, a reduction of renal deposition, and damage to the kidney. This may create a positive feedback loop to enhance renal excretion capacity. Evidence of intestinal-renal crosstalk has been observed [2] and is worth further exploration.

Bioinformatic analyses reveal the existence of multiple variants of the “reductive pathway” enzymes, which parallel earlier observations in the Pyd pathway, with *E. coli* XdhD and YgfK relying on NADH as the reductant, the corresponding *C. difficile* XdhD and UacX likely relying on Fdx as the reductant, and the gene cluster in *Chloroflexota* bacteria containing a putative yanthine reductase relying on coenzyme F420. Another key difference is the cleavage of the 2-ureido group through an irreversible hydrolytic reaction by *C. difficile* UacY, and a reversible phosphorylytic reaction by *E. coli* YgeW, forming carbamoyl phosphate to support ATP generation. These pathway variants may reflect the differences in redox mediators and energy conservation strategies. Further studies are needed to determine the optimal combination of alternative enzymes for engineering uricolytic probiotics, balancing UA flux and energy conservation.

## Limitations of the study

The computational models in this study revealed a putative role of Glu606 in determining the substrate specificity for XdhD−YgfM. Future studies using crystallography or cryo‑EM combined with site-directed mutagenesis are expected to unveil the detailed mechanism. In addition, although the uricase‑deficient mouse model reproduces many key features of hyperuricaemia, mice still differ from humans in purine metabolism, intestine length, and gut microbiota. Pharmacokinetic analyses and controlled clinical trials will be essential before translation.

## Materials and methods

### Strains, cultures, and chemicals

The strains and plasmids used are listed in Supplementary Tables S2 and S3, respectively. For UA utilization assays, *E. coli* cells were cultured anaerobically at 37°C in M9 minimal medium (1 × M9 salts, 2 mmol/L MgSO_4_, 0.1 mmol/L CaCl_2_, and 4 g/L glucose, pH 7.0) in an anaerobic vial, unless otherwise stated. For genetic manipulations, cells were grown aerobically in LB broth at 37°C. For oxygen-free cultivation, the medium was prepared in a Lab2000 glovebox (Etelux) under an atmosphere of N_2_ containing less than 5 ppm O_2_ by dissolving medium powder in degassed ddH_2_O, followed by filtration through a 0.2-μm filter. For bacteria used in mouse experiments, cells were inoculated into 5 mL LB broth and cultured overnight at 37°C with shaking. Cell growth in cultures was monitored by measuring optical density at 600 nm (OD_600_). All chemicals and reagents were of analytical grade and are detailed in Supplementary Table S4.

### Standard DNA manipulation

All oligonucleotides in this study were synthesized by Sangon Ltd. and are listed in Supplementary Table S5. DNA polymerases, T4 DNA ligases, and all restriction endonucleases were obtained from TaKaRa Bio Inc. and ThermoFisher Scientiﬁc Inc. Genomic DNAs and plasmid DNAs were extracted using the TIANamp Bacteria DNA Kit and the TIANprep Mini Plasmid Kit, respectively (Tiangen, China), while ampliﬁed PCR fragments were puriﬁed using the TIANquick Midi Puriﬁcation Kit (Tiangen, China).

Genes from *E. coli* MG1655 encoding *YgeW*, *YgeX*, *YgeY*, *HyuA*, *YqeA*, *YgfK*, and *SsnA* (Uniprot ID: Q46803, P66899, P65807, Q46806, Q46807, Q46811, and Q46812, respectively) were amplified by colony PCR and individually inserted into the pET28a-6*His-TEV (HT) vector [44]. Gene syntheses for *C. difficile*  *UacX* (Uniprot ID: Q181U4), *HyuA* (Uniprot ID: Q181U3), and *UacY* (Uniprot ID: Q181U6), and their expression were customized and inserted into the HT vector (Beijing Azenta Biotechnology Co., Ltd., China). The *xdhD* gene was cloned and expressed under its native promoters using the low-copy-number plasmid pSC101 (5−10 copies per cell) to minimize metabolic burden on the cofactor synthesis. The construct was generated by PCR amplification of the *xdhD* gene with its native promoter, followed by insertion of a TEV protease recognition site, a (GGGGS)*3 flexible linker, and a tandem Protein A (ProtA) tag at the C-terminus to facilitate affinity purification and activity assays [38]. Knockout mutants were generated via CRISPR-aided homologous recombination strategy and confirmed by sequencing [37, 45].

### Protein expression and purification

Typically, expression plasmids were transformed into *E. coli* BL21 (DE3) cells followed by induction with 0.3 mmol/L isopropyl β-D-1-thiogalactopyranoside (IPTG) at 18°C for 16 h. Cell pellets were harvested and resuspended in lysis buffer (50 mmol/L Tris-HCl, pH 8.0, 200 mmol/L KCl, 1 mmol/L PMSF, and 1 μL DNase I), and lysed using a pre-chilled high-pressure homogenizer. Cell debris was removed by centrifugation at 10,000 *g* for 10 min at 4°C. Streptomycin sulfate (final concentration, 2% (w/v)) was added to the cell lysate, followed by centrifugation to remove DNA. The supernatant was then applied to a 5-mL TALON Co²⁺ column (Cytiva, China) pre-equilibrated with buffer A (20 mmol/L Tris-HCl, pH 7.5, 200 mmol/L KCl, and 5 mmol/L β-mercaptoethanol [BME]). The column was washed with the same buffer and His-tagged proteins of interest were eluted with buffer B (20 mmol/L Tris-HCl, pH 7.5, 200 mmol/L KCl, 5 mmol/L BME, and 150 mmol/L imidazole), dialyzed against buffer A at 4°C for 3 h to remove imidazole, and concentrated using Amicon Ultra-15 centrifugal filters (Millipore, USA). The purified proteins were aliquoted and flash frozen in liquid nitrogen with 20% glycerol and stored at −80°C.

For XdhD (Q46814) expression and purification, IPTG induction was not necessary and was skipped. Cells from an overnight culture were lysed, followed by streptomycin sulfate precipitation. After centrifugation at 25,000 *g* for 60 min at 4°C, the clarified supernatant was applied to a 1-mL pre-equilibrated Rabbit IgG Beads 4FF column (Smart-Lifesciences Bio). The column was washed with 50 column volumes of lysis buffer, and on-column TEV protease cleavage (1 mg/mL) was performed at 4°C for 20 h to remove the ProtA tag. Finally, the protein was further purified using a HiLoad 16/600 Superdex 200 prep grade column equilibrated with size-exclusion chromatography buffer (20 mmol/L Tris-HCl, pH 8.0, 200 mmol/L KCl, and 1 mmol/L dithiothreitol [DTT]). The purified protein was then concentrated to 100 μL and stored at −80°C for subsequent enzymatic assays.

### Reconstitution and UV-Vis characterization of YgfK iron-sulfur (Fe-S) clusters

Reconstitution of Fe-S clusters and UV-Vis spectroscopic analyses were performed as previously described [2, 3]. Briefly, protein solutions were degassed under argon using a Schlenk line and subsequently transferred into an anaerobic glovebox. Reconstitution was carried out in a buffer containing 100 mmol/L Tris-HCl (pH 7.5), 10 mmol/L DTT, and 12 equivalents each of ferrous ammonium sulfate [(NH₄)₂Fe(SO₄)₂] and sodium sulfide (Na₂S·9H₂O). The mixtures were incubated at 4°C overnight, followed by the addition of 4 equivalents of EDTA, and removal of excess reagents by buffer exchange into 20 mmol/L Tris-HCl (pH 7.5) and 200 mmol/L KCl. UV-Vis absorption spectra (220–800 nm) of reconstituted *Ec*YgfK were recorded using a Nanophotometer NP80 Mobile (Implen, Germany). Samples were prepared at 10 μmol/L in anaerobic conditions and transferred into septum-sealed quartz cuvettes.

### *In vitro* enzymatic activity assays

To assess the catalytic activities of *Ec*XdhD−YgfM in the conversion of UA to yanthine, *in vitro* assays were conducted using freshly prepared UA (pH 7.5). Reactions were carried out in 20 mmol/L Tris-HCl (pH 7.5) containing 200 mmol/L KCl, 1 mmol/L NADH, 1 mmol/L UA, and 10 μmol/L enzyme at room temperature (RT).

For the YgeX/YgeY coupling assays, a reaction mixture of 5 mmol/L L/D-albizziin, 1 mmol/L CoCl₂, 10 μmol/L YgeX, and 10 μmol/L YgeY in 20 mmol/L Tris-HCl (pH 7.5) and 100 mmol/L KCl was incubated at 30°C for 60 min. For the *Cd*HyuA or *Cd*HyuA and *Cd*UacY coupling assays, 20 mmol/L Tris-HCl (pH 7.5), 100 mmol/L KCl, 10 mmol/L DOI-MU or 5UDU, 1 mmol/L ZnCl₂, and 10 μmol/L enzyme(s) were mixed and incubated at RT for 1 h. For the *Cd*HyuA and *Ec*YgeW coupling assays, 50 mmol/L PBS (pH 7.2), 100 mmol/L KCl, 10 mmol/L DOI-MU or 5UDU, 1 mmol/L MgCl₂, and 10 μmol/L enzyme(s) were mixed and incubated at RT for 1 h.

Assays involving YgfK/UacX and SsnA coupling reactions were performed in an anaerobic glovebox. For YgfK−SsnA coupling assays, reconstituted YgfK (10 μmol/L each) and degassed SsnA were mixed with 1 mmol/L yanthine, 1 mmol/L ZnCl_2_, 1 mmol/L NADH in 20 mmol/L Tris-HCl (pH 7.5), and 200 mmol/L KCl, and incubated at RT for 1 h. For UacX and SsnA coupling assays, typical enzymatic assays were performed in 200 μL reaction volumes containing 20 mmol/L Tris-HCl (pH 7.5), 200 mmol/L KCl, 0.5 mmol/L reduced methylviologen radical (MV•^+^), 0.35 mmol/L FMN, 1 mmol/L yanthine, and 10 μmol/L or 16.8 μmol/L reconstituted UacX. Reactions were incubated at 4°C for 2 h. Methylviologen (MV^2+^) was reduced to MV•^+^ using approximately 1.2 equivalents of titanium (III) citrate in an anaerobic glovebox. The concentration of MV•^+^ was determined spectrophotometrically at 600 nm using an extinction coefficient (ε_600_) of 13,700 L/(mol × cm). Controls omitting individual components, either enzyme or substrate, were included. All reactions were quenched by adding an equal volume of acetonitrile to precipitate proteins. Supernatants were collected after centrifugation and analyzed by LC-MS for product quantification.

### LC-MS analysis

Enzymatic reaction products were measured on an Agilent 1260 HPLC system coupled to an Agilent 6420 triple-quadrupole mass spectrometer. Separation was achieved on a ZIC-HILIC column (150 × 4.6 mm, 5 µm, 200 Å; Merck) using a linear gradient from 90% to 50% solvent B over 20 min at 0.75 mL/min. The mass spectrometer ran in positive-ion electrospray, multiple-reaction-monitoring mode (source temperature 330°C; drying-gas flow 10 L/min; nebuliser pressure 45 psi). Monitored transitions and collision energies were: UA (169.0/141.0, 15 eV), yanthine (153.0/110.0, 15 eV), UMH and 5UDU (173.0/130.0, 10 eV), and D/L-albizzin (148.0/88.1, 5 eV). Data were processed with Agilent MassHunter Qualitative Analysis software. HyuA, YgeX−YgeY, and HyuA−YgeW assay products were analyzed in full-scan mode.

### Growth conditions and sample preparation for metabolic intermediate analysis

*E. coli* cells were inoculated into 5 mL LB medium and cultured overnight at 37°C with shaking. Cells were harvested by centrifugation at 4,000 *g* for 10 min at 4°C, washed with sterile PBS, and resuspended in oxygen-free LB medium. Cell suspensions were diluted to an OD_600_ of 0.05 in 5 mL oxygen-free LB medium, supplemented with either UA (final 1.4 or 3.0 mmol/L) or yanthine (final 0.2 mmol/L), with or without 0.4% (w/v) glucose. At designated time points, aliquots of cultures were harvested by centrifugation (5,000 *g*, RT, 10 min). The supernatants were treated with alkaline solution (final approximately 2 mmol/L), centrifuged at 10,000 g for 10 min, filtered by 0.22-µm filter, and then analyzed by LC-MRM-MS to quantify xanthine, UA, yanthine, UMH, and albizziin. Data represent the mean ± standard error from three independent biological replicates.

### RNA extraction and sequencing

Cultures of CBT2.0 and WT strains were grown anaerobically in LB medium at 37°C, supplemented with 1 mmol/L yanthine. Cells were harvested at the logarithmic phase by centrifugation at 5,000 *g* for 10 min at 4°C. Total RNA was extracted using a commercial RNA isolation kit, and residual genomic DNA was removed by DNase treatment. RNA quality was assessed, and mRNA was enriched by depleting ribosomal RNA (rRNA). RNA library construction and high-throughput sequencing were performed by Novogene (China). Clean reads were aligned to the reference genome using Bowtie2 (v2.2.3). Gene-level read counts were obtained using HTSeq (v0.6.1) [46]. Differential gene expression analysis between CBT2.0 and WT strains cultured with yanthine was performed using DESeq2. Genes with adjusted *P* values (*P*_adj_) < 0.05 and |log_2_(fold change)| > 1 were considered significantly differentially expressed and were visualized in a volcano plot [47].

### Animal husbandry and sample collection

Six-week-old male uricase-deficient (*Uox*^*−/−*^) mice on a C57BL/6JGpt background (B6/JGpt-Uoxem3Cd3501/Gpt; GemPharmatech, China) were used. Mice were maintained on standard chow and allopurinol-supplemented water (100 mg/L) until 6 weeks of age, after which they were switched to normal drinking water. Animals had *ad libitum* access to food and water, and were housed under a 12-h light/12-h dark cycle at (20–22)°C with 40%–60% humidity. Bacterial suspensions containing CBT2.0 or WT strains were prepared at a density of 1 × 10^10^ cfu per 200 μL PBS. Mice were administered bacteria or PBS vehicle control daily by intragastric gavage over 6 weeks. Daily gavage administration was ceased at week 6, and mice were sacrificed at week 15 for the collection of plasma, colonic contents, and kidneys. Blood samples were collected from the caudal vein of live mice into tubes containing concentrated sodium EDTA (final concentration being approximately 12 mmol/L) and allopurinol (final concentration being approximately 12 µmol/L) to inhibit xanthine oxidase [48]. Plasma was obtained by centrifugation at 1,500 *g* for 10 min at 10°C. The plasma and colonic content samples were stored at −80°C until analysis, and kidney samples were fixed with 4% paraformaldehyde and stored at RT. The animal care and experimental protocols conformed to the recommendations in the 8th Edition of the Guide for the Care and Use of Laboratory Animals of the National Institutes of Health (NIH, revised 2011) and were in accordance with the Animal Management Rules of China (Documentation 55, 2001, Ministry of Health, China). Plasma UA, CRE, and urea levels were measured using commercial assay kits (Nanjing Jiancheng Bioengineering Institute; C012-2-1, C011-2-1, C013-2-1) according to the manufacturer’s instructions.

### Detection of *E. coli* CBT2.0

Genomic DNAs from colon contents were extracted using the TIANamp stool DNA kit (DP328-02, Tiangen, China) according to the manufacturer’s instructions. The success of DNA extraction was confirmed by A_260 nm_ measurement using a Nanophotometer NP80 (Implen, Germany). Genomic DNA samples were then subjected to RT-qPCR analysis. The RT-qPCR assays were performed on a real-time PCR system (SLAN96s, HONGSHI, China) using Universal SYBR Green Fast qPCR Mix (RK21203, ABclonal, China) staining. The average Ct value was calculated from triplicates or duplicates with a deviated data point (> 2 cycle differences) excluded from analysis. One sample in the WT group was excluded from the analysis due to contamination. The relative abundance of CBT2.0 was calculated as − (Ct (CBT2.0 specific fragment) − Ct (16S rDNA)) [49], and 45 was used when the Ct value was greater than 45. The primer sequences for CBT2.0-specific *gapA* and *nirB* promoters (2.0-Q-F/R) and 16S rDNA conserved region (16S-Q-F/R) were listed in Supplementary Table S5.

### Histological analysis of the kidney

The mouse kidney tissues were fixed with 4% paraformaldehyde for 48 h before further processing. The tissues were embedded in paraffin, and the sections were stained with H&E. Pathological analysis of the lungs was performed under a light microscope (Nikon, Eclipse Ci-L). The evaluation of kidney injury included neutrophil infiltration, cystic dilation, and tubular injury. The degree of neutrophil infiltration and cystic dilation was defined as mild, moderate, or severe. For neutrophil infiltration, semi-quantitative assessment was performed by counting the number of neutrophils under a high-power field (HPF): mild with 5–10 cells/HPF; moderate with 10–20 cells/HPF; severe with > 20 cells/HPF. The degree of cystic dilation was determined by the number and average size of kidney cysts. The degree of tubular injury was graded on a scale of 0–4 according to the severity of tubular vacuolation, tubular necrosis, and tubular dilatation (0: no change in the tubules; 1: ≤ 25% of tubular injury; 2: 25%–50% of tubular injury; 3: 50%–75% of tubular injury; 4: ≥ 75% of tubular injury).

### Clinical samples

In the first cohort study (Fig. 5h), with only five samples and the striking difference in yanthine contents between the two healthy individuals and three hyperuricemia patients, we did not perform statistical analysis. The second cohort consisted of a group of healthy individuals and a group of gout patients diagnosed by Tianjin First Central Hospital (Fig. 5i). Serum was obtained from 25 gout patients and 43 age- and sex-matched healthy volunteers under protocols approved by the Institutional Review Board of Tianjin First Central Hospital. After collection, the blood samples were allowed to clot at RT for 30 min and centrifuged at 1,500 *g* for 10 min at 4°C. Aliquots of serum (50 µL) were deproteinized by the addition of 150 µL ice-cold acetonitrile:MeOH (1:1, v/v), vortexed for 1 min, and centrifuged at 16,000 *g* for 10 min. The supernatants were analyzed on the Agilent 1260 HPLC–6420 triple-quadrupole platform under the MRM conditions described above. Serum UA and yanthine concentrations were quantified against external calibration curves.

### Quantification and statistical analysis

All statistical analyses and definitions of sample sizes are provided in the Figure legends. Unless otherwise indicated, experiments were performed with at least three biological replicates. Error bars in bar and line graphs represent the standard deviation (SD). For each sample, three to five independent experiments were conducted, each with three to five technical replicates. Statistical significance was assessed using Student’s unpaired *t*-test. *P* value < 0.05 was considered statistically significant. ^*^P < 0.05; ^**^P < 0.01; ^***^P < 0.001.

### Bioinformatics analysis

To construct the SSN of the SsnA family (IPR017700) in bacteria, a non-redundant sequence set was assembled by downloading all UniProt entries annotated with the “SsnA family” domain (IPR017700) and mapping them to their corresponding UniRef90 clusters (proteins clustered at ≥ 90% sequence identity), excluding fragment sequences. An SSN was generated in EFI-EST (Enzyme Function Initiative-Enzyme Similarity Tool) [50] using an edge E-value cutoff of ≤ 10^−140^. Genome-neighborhood information within a 15-ORF window was retrieved with EFI-GNT (Enzyme Function Initiative-Genome Neighborhood Tool) [50] to detect co-occurrence with selected protein families. The network was visualized in Cytoscape [51]. Nodes are colored by phylum or presence of specific protein families within 15-ORF neighborhoods. These include XdhA/XdhD homologs (some with YgfM FAD domain) and either YgfK or UacX. *Chloroflexota* lack these but have a putative F420-dependent “luciferase-family” dehydrogenase, suggesting an alternative xanthine reduction pathway. SsnA frequently colocalizes with downstream pathway genes *HyuA*, *YgeW/UacY*, *YgeY*, and *YgeX*.

## Supplementary Material

loaf031_suppl_Supplementary_Materials

## Data Availability

All data needed to evaluate the conclusions in the article are present in the article and/or the Supplementary data.
