# Supplementary material for: A reductive uric acid degradation pathway in anaerobic bacteria
Source: Life Metab. 2025 Jul 31;4(6):loaf031. doi: 10.1093/lifemeta/loaf031 (PMC12507026; doi:10.1093/lifemeta/loaf031)
Supplement: loaf031_suppl_Supplementary_Materials [file loaf031_suppl_supplementary_materials.docx]

**Supplementary Information for**

**A reductive uric acid degradation pathway in anaerobic bacteria**

Zhi Li^1,‡^, Wei Meng^1,‡^, Zihan Gao^2,‡^, Wanli Peng^2^, Zhandong Hu^3^, Jianhao Zhang^2^, Yining Wang^1^, Xiaoxia Wu^1^, Zipeng Zhao^1^, Chuyuan Zhang^1^, Zhuohao Tang^2^, Zhujun Nie^1^, Shaohua Wu^3^, Benjuan Wu^3^, Hui Zheng^4^, Duqiang Luo^5^, Yang Tong^1^, Yiling Hu^1^, Zehan Hu^2^, Yifeng Wei^6,*^, Yan Zhang^1,2,7,8,*^

^1^New Cornerstone Science Laboratory, School of Pharmaceutical Science and Technology, Tianjin University, Tianjin 300072, China

^2^School of Life Sciences and Biotechnology, Shanghai Jiao Tong University, Shanghai 200240, China

^3^Tianjin First Central Hospital, Tianjin 300190, China

^4^Department of Anesthesiology, National Cancer Center, Chinese Academy of Medical Sciences and Peking Union Medical College, Beijing 100021, China

^5^College of Life Sciences, Institute of Life Science and Green Development and Hebei Innovation Center for Bioengineering and Biotechnology, Hebei University, Baoding, Hebei 071002, China

^6^Singapore Institute of Food and Biotechnology Innovation (SIFBI), Agency for Science, Technology and Research (A*STAR), Singapore 138669, Singapore

^7^Frontiers Science Center for Synthetic Biology (Ministry of Education), Tianjin University, Tianjin 300072, China

^8^Key Laboratory of Systems Bioengineering (Ministry of Education), School of Chemical Engineering and Technology, Tianjin University, Tianjin 300072, China

^‡^These authors contributed equally to this work.

**^*^**Corresponding authors. Singapore Institute of Food and Biotechnology Innovation (SIFBI), Agency for Science, Technology and Research (A*STAR), Singapore 138669, Singapore. E-mail: [wei_yifeng@sifbi.a-star.edu.sg](mailto:wei_yifeng@sifbi.a-star.edu.sg) (Y.W.); New Cornerstone Science Laboratory, School of Pharmaceutical Science and Technology, Tianjin University, Tianjin 300072, China. E-mail: yan.zhang@tju.edu.cn

**Supplementary Tables S1−S6; Supplementary Figures S1−S8.**

**Supplementary Table S1** Comparison of renal injury scores between control and CBT2.0-treated mice.

| **Group** | **Number** | **Degree of neutrophil infiltration** | **Degree of cystic dilation** | **Number of cystic dilations** | **Tubular**  **injury score** | **Mean 2.0sp Ct** | **Mean 16s Ct** | **ΔCt** | **UA level in plasma (μmol/L)** |
| --- | --- | --- | --- | --- | --- | --- | --- | --- | --- |
| CBT2.0 | 1 | Mild | Mild | 3 | 1 | 34.65 | 13.39 | 21.27 | 319 |
|  | 2 | Severe | Mild | 3 | 2 | > 45 | 16.03 | 28.98 | 379 |
|  | 3 | Mild | Mild | 3 | 1 | 31.05 | 12.62 | 18.43 | 250 |
|  | 4 | Mild | Moderate | 7 | 1 | > 45 | 16.00 | 29.00 | 371 |
|  | 5 | Mild | - | 0 | 1 | 35.71 | 10.99 | 24.73 | 218 |
|  | 6 | Mild | Mild | 5 | 1 | 41.59 | 12.59 | 29.00 | 430 |
| WT | 1 | Moderate | Severe | 3 | 2 | - | 13.22 | - | 411 |
|  | 2 | Severe | Severe | 8 | 3 | > 45 | 14.39 | 30.61 | 478 |
|  | 3 | Moderate | Moderate | 7 | 2 | > 45 | 12.39 | 32.61 | 380 |
|  | 4 | Mild | Moderate | 8 | 1 | > 45 | 15.40 | 29.60 | 424 |
|  | 5 | Moderate | Severe | 13 | 3 | > 45 | 12.59 | 32.41 | 533 |
|  | 6 | Moderate | Severe | 10 | 2 | > 45 | 14.62 | 30.38 | 701 |
| PBS | 1 | Moderate | Severe | 6 | 2 | > 45 | 13.30 | 31.70 | 430 |
|  | 2 | Severe | Moderate | 7 | 2 | > 45 | 10.70 | 34.30 | 456 |
|  | 3 | Severe | Moderate | 8 | 2 | > 45 | 13.10 | 31.90 | 508 |
|  | 4 | Moderate | Severe | 12 | 2 | > 45 | 12.51 | 32.49 | 459 |
|  | 5 | Severe | Severe | 8 | 2 | > 45 | 13.38 | 31.62 | 484 |
|  | 6 | Severe | Moderate | 12 | 2 | 43.85 | 11.19 | 32.66 | 427 |

**Supplementary Table S2** Strains used in this study.

| **Strains** | **Description** | **Reference/source** |
| --- | --- | --- |
| *E. coli* MG1655 | Coli Genetic Stock Center strain (CGSC) No. 6300 | CGSC |
| *E. coli* Nissle 1917 | Coli Genetic Stock Center strain | CGSC |
| *E. coli* MG1655 Δ*xdhA* | The *xdhA*-deleted mutant of *E. coli* MG1655 | This study |
| *E. coli* MG1655 Δ*xdhD* | The *xdhD*-deleted mutant of *E. coli* MG1655 | This study |
| *E. coli* Nissle 1917 Δ*xdhA* | The *mdtL*-deleted mutant of *E. coli* Nissle 1917 | This study |
| *E. coli* Nissle 1917 Δ*xdhD* | The *mdtG*-deleted mutant of *E. coli* Nissle 1917 | This study |
| *E. coli* Nissle 1917 Δ*ygfK* | The *ygfK*-deleted mutant of *E. coli* Nissle 1917 | This study |
| CarBT4gout 2.0 strain | The *_p_gapA-_p_nirBp* insertion of *E. coli* Nissle 1917 | This study |
| CarBT4gout 2.0 strain; Δ*xdhA* | The *xdhA*-deleted mutant of *E. coli* CarBT4gout 2.0 strain | This study |
| CarBT4gout 2.0 strain; Δ*xdhD* | The *xdhD*-deleted mutant of *E. coli* CarBT4gout 2.0 strain | This study |

**Supplementary Table S3** Plasmids used in this study.

| **Plasmids** | **Description** | **Source/reference** |
| --- | --- | --- |
| HT-YgeW | for *Ec*YgeW expression | This study |
| HT-YgeX | for *Ec*YgeX expression | This study |
| HT-YgeY | for *Ec*YgeY expression | This study |
| HT-HyuA | for *Ec*HyuA expression | This study |
| HT-YqeA | for *Ec*YqeA expression | This study |
| HT-YgfK | for *Ec*YgfK expression | This study |
| HT-SsnA | for *Ec*SsnA expression | This study |
| HT-*Cd*HyuA | for *Cd*HyuA expression | This study |
| HT-*Cd*UacX | for *Cd*UacX expression | This study |
| HT*-Cd*UacY | for *Cd*UacY expression | This study |
| pSC101 | Vector with a low-copy replication pSC101 origin, Km^R^ | Novagen |
| pSC101-XdhD | The plasmid pSC101 inserted with proteinA labeled *xdhD* gene, Km^R^ | This study |
| pCas | Vector for genome editing, pSC101 origin, Km^R^ | [1] |
| pTargetF | Vector for genome editing, pMB1 origin, Sm^R^ | [1] |
| pTarget-XdhA | pTargetF plasmid harboring correspond sgRNA, with donor DNA fragment at the *Hind*III/*Xho*I sites, Sm^R^ | This study |
| pTarget-XdhD | pTargetF plasmid harboring correspond sgRNA, with donor DNA fragment at the *Hind*III/*Xho*I sites, Sm^R^ | This study |

**Supplementary Table S4** Chemicals used in this study.

| **Chemicals and reagents** | **Source** | **Identifier** |
| --- | --- | --- |
| Acetonitrile, HPLC grade | MACKLIN | A800362-4L |
| Agar gel strength | Solarbio | A8191-500g |
| Allopurinol | MERYER | M13658-500G |
| Ammonium acetate | MERYER | M22256-100G |
| Ampicillin | BBI | A610028-0025 |
| Calcium chloride dihydrate | Greagent | G10224B |
| Creatinine | Shanghai Yuanye Bio-Technology | B25917-100mg |
| Creatinine (N-methyl-D3, 98%) | Shanghai Yuanye Bio-Technology | T19668-25mg |
| DTT (dithiothreitol) | BBI | A620058-0005 |
| Ethanol | MERYER | F30022-4L |
| Glycerol | BBI | A450039-0500 |
| Hypoxanthine | Sangon Biotech | A500336-0050 |
| Kanamycin sulfate | Shanghai Yuanye Bio-Technology | S17025-100g |
| L-albizziin | MCE | HY-121167 |
| L-cysteine | BBI | A600132-0100 |
| L-(+)-arabinose | Adamas | 63128D |
| Lysozyme | Solarbio | L8120 |
| M9 minimal salts | BBI | A507024-0250 |
| Magnesium sulfate heptahydrate | Greagent | G10221C |
| Methanol | MACKLIN | M813903-4L |
| PMSF (phenylmethanesulfonyl fluoride) | Shanghai Yuanye Bio-Technology | S30438 |
| Sodium bicarbonate | Aladdin | S112331-500g |
| Sodium chloride | Greagent | G81793J |
| Sodium hydroxide | Aladdin | S111507-500g |
| Spectinomycin dihydrochloride pentahydrate | BBI | A600901-0005 |
| Tris | Shanghai Yuanye Bio-Technology |  |
| Tris-HCl | MERYER |  |
| Tryptone | BBI | A650217-0500 |
| Uric acid | MERYER | M23894-25G |
| Uric acid-[1,3-^15^N_2_] | Shanghai Yuanye Bio-Technology | B45070-10mg |
| Water | Millipore | Milli-Q® SQ 2 |
| Xanthine | MERYER | M23876-25G |
| Yanthine (2,8-dioxopurine) | Shanghai Yuanye Bio-Technology | Y90960-1g |
| Yeast extract | OXOID | LP0021B |

**Supplementary Table S5** Oligonucleotides used in this study.

| **Name** | **Sequences (5’-3’)** | **Description** |
| --- | --- | --- |
| pTarget-VF | AAGCTTCTGCAGGTCGACTC | Used for construction of pTargetF series plasmids |
| pTarget-VR | CTCGAGTTCATGTGCAGCTCC |  |
| XdhA-UF | GACCTGCAGAAGCTTGAGCCGGAATACGTTCCTGT | Used for the construction of plasmid pT-XdhA |
| XdhA-UR | ATGTTATCCAGAAATCCCCTCGGTTTGAACATTT |  |
| XdhA-DR | GGGATTTCTGGATAACATCATGTTTGATTTTGCTTCTTACCA | Used for the construction of plasmid pT-XdhA |
| XdhA-DR | GCACATGAACTCGAGGCGGAAAATGAAAGGCGACA |  |
| XdhD-UF | GACCTGCAGAAGCTTTCAGTCGACCATTGGTGGTG | Used for the construction of plasmid pT-XdhD |
| XdhD-UR | ATTTATTACACGGCTTCCTCCCCAGC |  |
| XdhD-DR | GGAAGCCGTGTAATAAATGGCCGCTTCGGC | Used for the construction of plasmid pT-XdhD |
| XdhD-DR | GCACATGAACTCGAGACAACCAGAAATGCGCCAAC |  |
| pSC101-VF | TGCAGCTCCACGTAAATGCATGCCGCTTC | Used for construction of pTargetF series plasmids |
| pSC101-VR | GGTCGACTCACTCTGACTGCAAACCCTGC |  |
| XdhA-F | GTCAGAGTGAGTCGACCTGCAGAAGCTT | Used for the construction of plasmid pSC101-XdhA |
| XdhA-R | ATTTACGTGGAGCTGCACATGAACTCGA |  |
| XdhD-F | GTCAGAGTGAGTCGACCTGCAGAAGCTT | Used for the construction of plasmid pSC101-XdhD |
| XdhD-R | ATTTACGTGGAGCTGCACATGAACTCGA |  |
| HyuA-F | GAAAACCTGTACTTCCAATCCAATATGCGCGTATTGATCAAAAACGG | Used for the construction of plasmid HT-HyuA |
| HyuA-R | CGGATCCGTTATCCACTTCCAATTTAGAGCACGGGAGGGACAAAC |  |
| SsnA-F | TCCAATCCAATATGTTGATTCTGAAGAATGTCACTGC | Used for the construction of plasmid HT-SsnA |
| SsnA-R | ACTTCCAATTTATGCCAGCGCATCCATCC |  |
| XdhD-F | GAAAACCTGTACTTCCAATCCAATATGATCATCCACTTTACTTTAAATGGCG | Used for the construction of plasmid HT-XdhD |
| XdhD-R | CGGATCCGTTATCCACTTCCAATTTATATTTTTTCCAGCGCAGTGAG |  |
| YgeW-F | GAAAACCTGTACTTCCAATCCAATATGATGAAAACTGTTAATGAGCTGATTAAGG | Used for the construction of plasmid HT-YgeW |
| YgeW-R | CGGATCCGTTATCCACTTCCAATTTATTTCACGCGTTCTTGCGC |  |
| YgeX-F | GAAAACCTGTACTTCCAATCCAATATGTCCGTTTTCTCATTGAAGATTGATATCG | Used for the construction of plasmid HT-YgeX |
| YgeX-R | CGGATCCGTTATCCACTTCCAATTTAAGGTGCTACAGCGTGTTTG |  |
| YgeY-F | GAAAACCTGTACTTCCAATCCAATATGGCTAAGAATATTCCATTCAAACTGATTCTTG | Used for the construction of plasmid HT-YgeY |
| YgeY-R | CGGATCCGTTATCCACTTCCAATTTACTCTGTTGCCAGCCAGCTTA |  |
| YgfK-F | GAAAACCTGTACTTCCAATCCAATATGGGGGATATTATGCGTCCCATTCC | Used for the construction of plasmid HT-YgfK |
| YgfK-R | GGATCCGTTATCCACTTCCAATTTACACCTCCACGCGGCCCAG |  |
| YgfM-F | TCCAATCCAATATGATTGAACAATTTTTCAGGCCC | Used for the construction of plasmid HT-YgfM |
| YgfM-R | CTTCCAATTTATACGGCTTCCTCCCCAG |  |
| HT-F | TAAATTGGAAGTGGATAACGGATCCG | Used for the construction of plasmid HT-Gene |
| HT-R | CATATTGGATTGGAAGTACAGGTTTTCA |  |
| T7 | TAATACGACTCACTATAGG | Sequencing primers for ht plasmid construct verification |
| T7-Ter | GCTAGTTATTGCTCAGCGG |  |
| XdhA-U-F | AGTAGAAACAGACGTTCGGATGATTTGCTACCCGA | Used for the construction of plasmid pCas9-XdhA |
| XdhA-U-R | GTTATCCAGAAATCCCCTCGATTTGAACATTT |  |
| XdhA-D-F | GATTTCTGGATAACATCATGTTTGATTTTGCTTCT | Used for the construction of plasmid pCas9-XdhA |
| XdhA-D-R | TTCTAGCGTCATGCTCAAGAGACACT |  |
| Cas9- XdhA-U-F | GAACAAGCCAGAAGTTTGCCGTTTTAGAGCTA  GAAATAGCAAGTTAAAAT | Used for the construction of plasmid pCas9-XdhA |
| Cas9- XdhA-U-R | ATCATCCGAACGTCTGTTTCTACTGGTATTGGC |  |
| Cas9- XdhA-D-F | ATGACGCTAGAAGCTTGGATTCTCACCAATAAAA | Used for the construction of plasmid pCas9-XdhA |
| Cas9- XdhA-D-R | GGCAAACTTCTGGCTTGTTCCTAAGATC  TGACTCCATAACAGAGTAC |  |
| XdhD-U-F | AGTAGAAACAGACGTTACTCACGCCATGTTCGTAA | Used for the construction of plasmid pCas9-XdhD |
| XdhD-U-R | ATTTATTATACGGCTTCCTCCCCAG |  |
| XdhD-D-F | GAAGCCGTATAATAAATGGCCGCTTCGG | Used for the construction of plasmid pCas9-XdhD |
| XdhD-D-R | CCAAGCTTCTAGAACCGTCGCTTTTCATGC |  |
| Cas9- XdhD-U-F | CCGCAGAATCTCTGGGCAAAGTTTTAGAGCT  AGAAATAGCAAGTTAAAAT | Used for the construction of plasmid pCas9-XdhD |
| Cas9- XdhD-U-R | ACGAACATGGCGTGAGTAACGTCTGTTTCT  ACTGGTATTGGC |  |
| Cas9- XdhD-D-F | AAAGCGACGGTTCTAGAAGCTTG  GATTCTCACCAATAA | Used for the construction of plasmid pCas9-XdhD |
| Cas9- XdhD-D-R | TTTGCCCAGAGATTCTGCGGCTAA  GATCTGACTCCATAACAGAGTAC |  |
| YgfK-U-F | AGTAGAAACAGACGTAACCATGCCTGATGCAGG | Used for the construction of plasmid pCas9-YgfK |
| YgfK-U-R | CAACATGAAACCCTCTCCTTAGCTTATACAG |  |
| YgfK-D-F | TAAGGAGAGGGTTTCATGTTGATTCTGAAGAATGTCACTG | Used for the construction of plasmid pCas9-YgfK |
| YgfK-D-R | CTTCTAGAAATGCGTCGCGCAATG |  |
| Cas9-YgfK-U-F | AATTCTTGACCGCCTGGAGCGTTTTAGAGCTAGAAATAGCAAGTTAAAAT | Used for the construction of plasmid pCas9-YgfK |
| Cas9-YgfK-U-R | CCTGCATCAGGCATGGTTACGTCTGTTTCTACTGGTATTGGCAC |  |
| Cas9-YgfK-D-F | CGCGACGCATTTCTAGAAGCTTGGATTCTCACCAATAAAAAACGC | Used for the construction of plasmid pCas9-YgfK |
| Cas9-YgfK-D-R | GCTCCAGGCGGTCAAGAATTCTAAGATCTGACTCCATAACAGAGTAC |  |
| gRNA-test-F | CGAACTCAACGCCGGAT | Used for the gRNA test of plasmid pCas9 |
| gRNA-test-R | CGAAGGTGAGCCAGTGTG |  |
| homology-test-F | ACAGCGGCTGGCTGAGGAG | Used for the region of homology test of plasmid pCas9 |
| homology-test-R | CTCCATCTGGATTTGTTCAGAACGC |  |
| XdhA-test-F | TCGGATGATTTGCTACCCGAA | Used for the region of ΔXdhA test of *E. coli* strain |
| XdhA-test-R | CGTCATGCTCAAGAGACAC |  |
| XdhD-test-F | TACTCACGCCATGTTCGTAAT | Used for the region of ΔXdhD test of *E. coli* strain |
| XdhD-test-R | AACCGTCGCTTTTCATGC |  |
| YgfK-test-F | AACCATGCCTGATGCAGG | Used for the region of ΔYgfK test of *E. coli* strain |
| YgfK-test-F | AAATGCGTCGCGCAATG |  |
| gapA-F | ATATGTGATCAAATATTCCACCAGCTATTTGTTAGTG | Used for the construction of CarBT4gout 2.0 |
| gapA-R | AAACCCGCCGAAGCGGGTTTTTTCGAAAATTGTCACTTTGCTCACATCTCACTTTAATCG |  |
| nirB-F | TCGGCGGGTTTTTTTATAGCTAAAACAGGAGGTATATAAAGGTGAATTTGATTTACATCA | Used for the construction of CarBT4gout 2.0 |
| nirB-R | CGGCTGCGAAAGGCTTCTATTACCGCCTACCTTAACG |  |
| Cas9-gapA-U-F | AATTTGACGGATAATGTGTGGTTTTAGAGCTAGAAATAGCAAGTTAAAAT | Used for the construction of CarBT4gout 2.0 |
| Cas9-gapA-U-R | ATGATCGAACGTCTGTTTCTACTGGTATTGGC |  |
| gapAnirB-F | AAACAGACGTTCGATCATGGTCGCGAATATTTTAC | Used for the construction of CarBT4gout 2.0 |
| gapAnirB-R | GTGAGAATCCAAGCTTCTAGGCGAATACCGATAGCGTCAGCG |  |
| Cas9-gapA-D-F | ATCGGTATTCGCCTAGAAGCTTGGATTCTCACCAATAAAAAACGC | Used for the construction of CarBT4gout 2.0 |
| Cas9-gapA-D-R | CACACATTATCCGTCAAATTCTAAGATCTGACTCCATAACAGAGTAC |  |
| tssKO-test-F | GCTGTCTGATTAACAAACTGAACCC | Used for the _p_gapA__p_nirB insertion test of CarBT4gout 2.0 |
| tssKO-test-R | GGTCAATATCGCATTGCAGGTTC |  |
| EcNXdhA-U-F | GACCTGCAGAAGCTTGAGCCGGAATACGTTCCTGT | Used for the construction of plasmid pCas9-ΔXdhA |
| EcNXdhA-U-R | ATGTTATCCAGAAATCCCCTCGGTTTGAACATTT |  |
| EcNXdhA-D-F | GGGATTTCTGGATAACATCATGTTTGATTTTGCTTCTTACCA | Used for the construction of plasmid pCas9-ΔXdhA |
| EcNXdhA-D-R | GCACATGAACTCGAGGCGGAAAATGAAAGGCGACA |  |
| EcNXdhD-U-F | GACCTGCAGAAGCTTTCAGTCGACCATTGGTGGTG | Used for the construction of plasmid pCas9-ΔXdhD |
| EcNXdhD-U-R | ATTTATTACACGGCTTCCTCCCCAGC |  |
| EcNXdhD-D-F | GGAAGCCGTGTAATAAATGGCCGCTTCGGC | Used for the construction of plasmid pCas9-ΔXdhD |
| EcNXdhD-D-R | GCACATGAACTCGAGACAACCAGAAATGCGCCAAC |  |
| 2.0-Q-F | GCTTGAGACCACATGGGGTC | Used to detect CarBT4gout 2.0 colonization |
| 2.0-Q-R | CTATTACCGCCTACCTTAACGATTCAG |  |
| 16S-Q-F | GCAGGCCTAACACATGCAAGTC |  |
| 16S-Q-R | CTGCTGCCTCCCGTAGGAGT |  |

**Supplementary Table S6** Plasma uric acid, urine, creatinine, and CarBT4gout 2.0 colonization in *Uox^−/−^* mice.

| **Plasma uric acid, urine, creatinine and CarBT4gout 2.0 colonization in *Uox^−/−^* mice** | | | | | |
| --- | --- | --- | --- | --- | --- |
| Time point | Treatment | Mouse number | UA (μmol/L) | UN（mmol/L） | CRE (μmol/L) |
| Gavaged for 0 week | 2.0 | 1 | 315 | 50 | 60 |
|  |  | 2 | 352 | 23 | 42 |
|  |  | 3 | 459 | 79 | 58 |
|  |  | 4 | 384 | 75 | 56 |
|  |  | 5 | 163 | 18 | 36 |
|  |  | 6 | 223 | 45 | 62 |
|  | WT | 1 | 132 | 50 | 209 |
|  |  | 2 | 228 | 50 | 40 |
|  |  | 3 | 197 | 48 | 42 |
|  |  | 4 | 360 | 57 | 42 |
|  |  | 5 | 324 | 58 | 70 |
|  |  | 6 | 589 | 42 | 80 |
|  |  | 7 | 374 | 56 | 25 |
|  | PBS | 1 | 324 | 42 | 64 |
|  |  | 2 | 371 | 58 | 52 |
|  |  | 3 | 334 | 48 | 106 |
|  |  | 4 | 350 | 37 | 56 |
|  |  | 5 | 311 | 56 | 50 |
|  |  | 6 | 389 | 50 | 76 |
| Gavaged for 1 week | 2.0 | 1 | 204 | 40 |  |
|  |  | 2 | 313 | 34 |  |
|  |  | 3 | 360 | 35 |  |
|  |  | 4 | 593 | 36 |  |
|  |  | 5 | 133 | 22 |  |
|  |  | 6 | 405 | 42 |  |
|  | WT | 1 | 330 | 43 |  |
|  |  | 2 | 349 | 32 |  |
|  |  | 3 | 427 | 34 |  |
|  |  | 4 | 389 | 59 |  |
|  |  | 5 | 325 | 36 |  |
|  |  | 6 | 738 | 64 |  |
|  |  | 7 | 744 | 82 |  |
|  | PBS | 1 | 474 | 22 |  |
|  |  | 2 | 598 | 42 |  |
|  |  | 3 | 410 | 37 |  |
|  |  | 4 | 514 | 38 |  |
|  |  | 5 | 533 | 38 |  |
|  |  | 6 | 441 | 39 |  |
| Gavaged for 2 weeks | 2.0 | 1 | 287 | 34 |  |
|  |  | 2 | 313 | 39 |  |
|  |  | 3 | 364 | 35 |  |
|  |  | 4 | 279 | 33 |  |
|  |  | 5 | 293 | 19 |  |
|  |  | 6 | 397 | 51 |  |
|  | WT | 1 | 351 | 46 |  |
|  |  | 2 | 437 | 36 |  |
|  |  | 3 | 284 | 36 |  |
|  |  | 4 | 440 | 61 |  |
|  |  | 5 | 362 | 51 |  |
|  |  | 6 | 463 | 55 |  |
|  |  | 7 | 304 | 49 |  |
|  | PBS | 1 | 363 | 34 |  |
|  |  | 2 | 414 | 38 |  |
|  |  | 3 | 379 | 40 |  |
|  |  | 4 | 389 | 45 |  |
|  |  | 5 | 326 | 43 |  |
|  |  | 6 | 335 | 45 |  |
| Gavaged for 3 weeks | 2.0 | 1 | 244 | 29 | 28 |
|  |  | 2 | 154 | 43 | 31 |
|  |  | 3 | 106 | 35 | 26 |
|  |  | 4 | 184 | 28 | 37 |
|  |  | 5 | 78 | 18 | 30 |
|  |  | 6 | 176 | 38 | 37 |
|  | WT | 1 | 228 | 30 | 30 |
|  |  | 2 | 209 | 27 | 26 |
|  |  | 3 | 195 | 31 | 30 |
|  |  | 4 | 312 | 36 | 35 |
|  |  | 5 | 190 | 32 | 30 |
|  |  | 6 | 298 | 43 | 40 |
|  |  | 7 | 239 | 36 | 25 |
|  | PBS | 1 | 309 | 37 | 16 |
|  |  | 2 | 233 | 42 | 40 |
|  |  | 3 | 241 | 43 | 38 |
|  |  | 4 | 230 | 50 | 28 |
|  |  | 5 | 171 | 42 | 43 |
|  |  | 6 | 312 | 40 | 21 |
| Gavaged for 4 weeks | 2.0 | 1 | 173 | 16 |  |
|  |  | 2 | 339 | 28 |  |
|  |  | 3 | 223 | 15 |  |
|  |  | 4 | 450 | 35 |  |
|  |  | 5 | 47 | 48 |  |
|  |  | 6 | 202 | 48 |  |
|  | WT | 1 | 426 | 49 |  |
|  |  | 2 | 308 | 39 |  |
|  |  | 3 | 342 | 47 |  |
|  |  | 4 | 618 | 42 |  |
|  |  | 5 | 366 | 43 |  |
|  |  | 6 | 363 | 41 |  |
|  |  | 7 | 434 | 36 |  |
|  | PBS | 1 | 352 | 37 |  |
|  |  | 2 | 266 | 54 |  |
|  |  | 3 | 405 | 39 |  |
|  |  | 4 | 397 | 42 |  |
|  |  | 5 | 466 | 34 |  |
|  |  | 6 | 476 | 41 |  |
| Gavaged for 5 weeks | 2.0 | 1 | 282 | 23 |  |
|  |  | 2 | 367 | 19 |  |
|  |  | 3 | 300 | 16 |  |
|  |  | 4 | 294 | 19 |  |
|  |  | 5 | 142 | 9 |  |
|  |  | 6 | 317 | 19 |  |
|  | WT | 1 | 241 | 23 |  |
|  |  | 2 | 481 | 23 |  |
|  |  | 3 | 273 | 22 |  |
|  |  | 4 | 446 | 31 |  |
|  |  | 5 | 370 | 23 |  |
|  |  | 6 | 361 | 23 |  |
|  |  | 7 | 247 | 26 |  |
|  | PBS | 1 | 460 | 22 |  |
|  |  | 2 | 490 | 28 |  |
|  |  | 3 | 498 | 24 |  |
|  |  | 4 | 735 | 29 |  |
|  |  | 5 | 484 | 25 |  |
|  |  | 6 | 320 | 25 |  |
| Gavaged for 6 weeks | 2.0 | 1 | 113 | 35 | 16 |
|  |  | 2 | 300 | 42 | 16 |
|  |  | 3 | 186 | 27 | 13 |
|  |  | 4 | 186 | 39 | 17 |
|  |  | 5 | 31 | 19 | 8 |
|  |  | 6 | 215 | 39 | 19 |
|  | WT | 1 | 338 | 35 | 23 |
|  |  | 2 | 253 | 40 | 29 |
|  |  | 3 | 212 | 40 | 22 |
|  |  | 4 | 884 | 63 | 19 |
|  |  | 5 | 159 | 47 | 28 |
|  |  | 6 | 624 | 49 | 20 |
|  |  | 7 | 244 | 34 | 26 |
|  | PBS | 1 | 574 | 43 | 32 |
|  |  | 2 | 472 | 55 | 41 |
|  |  | 3 | 381 | 50 | 38 |
|  |  | 4 | 510 | 53 | 34 |
|  |  | 5 | 411 | 44 | 22 |
|  |  | 6 | 431 | 48 | 37 |


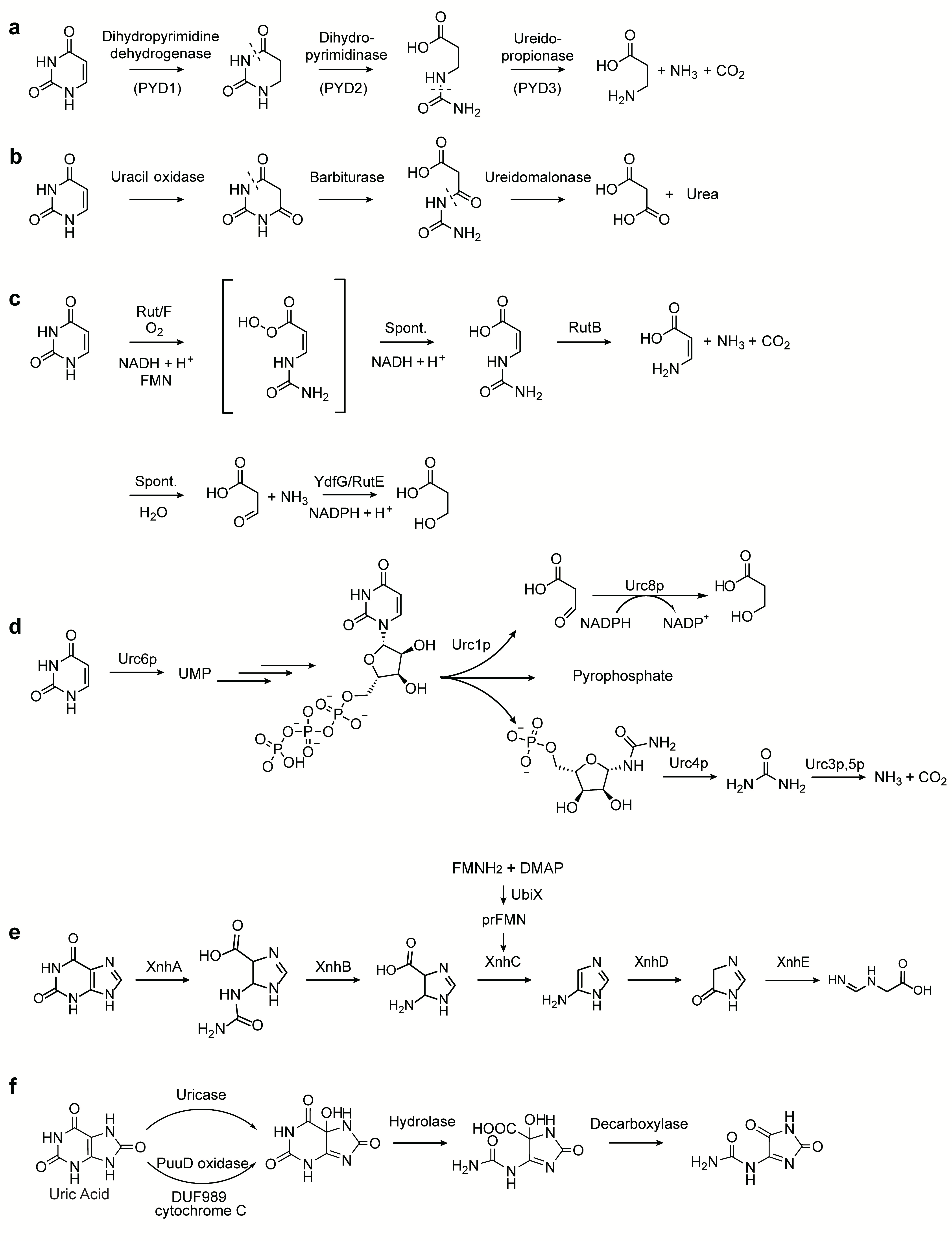


**Supplementary Figure S1** Pyrimidine and purine degradation pathway. (a) Pyd pathway. (b) Oxidative pyrimidine degradation pathway. (c) Rut pathway. (d) URC pathway in *Lachancea kluyveri.* (e) Xanthinase pathway. (f) Oxidative uric acid degradation pathway.


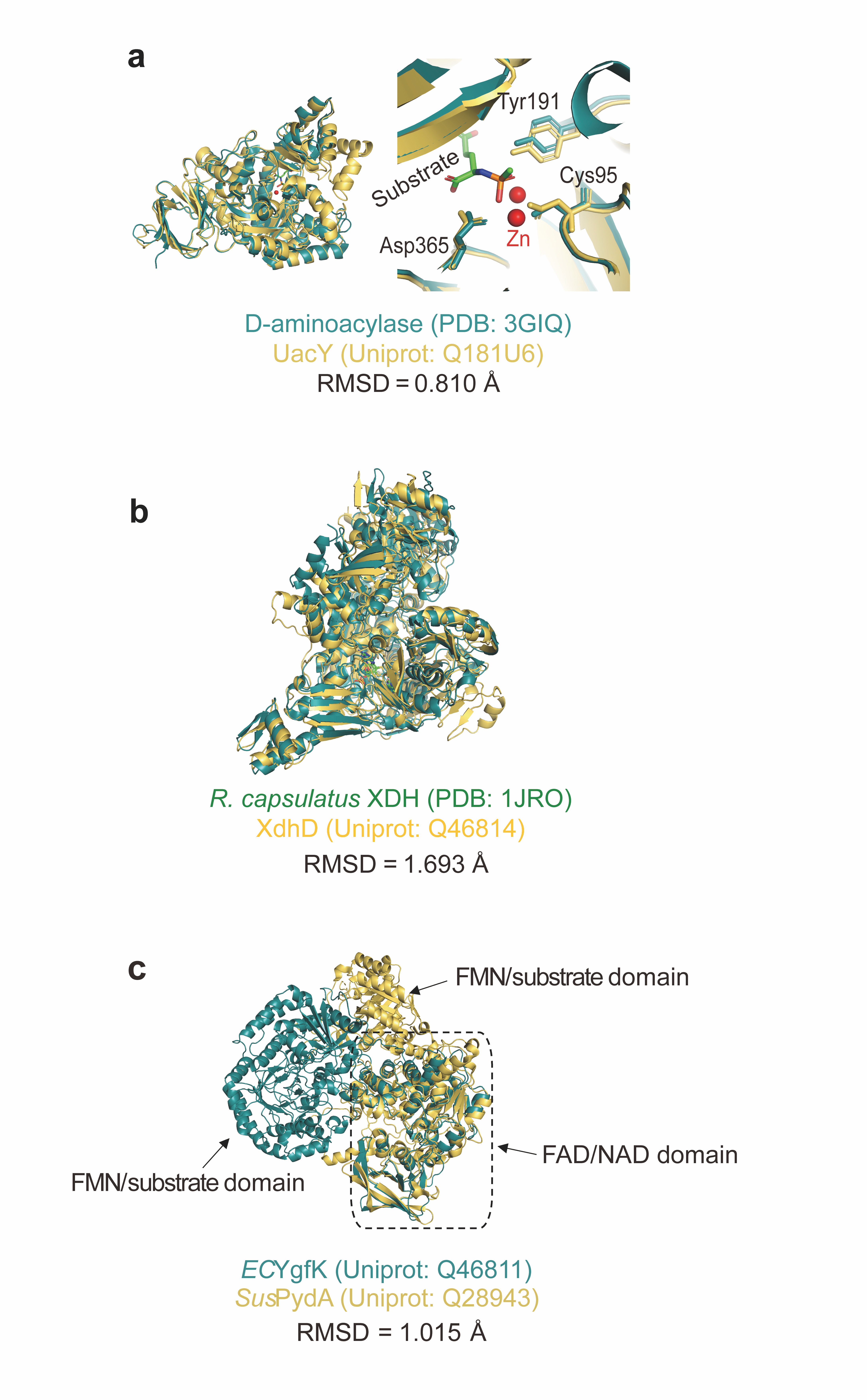


**Supplementary Figure S2** Structural comparisons of UacY, XdhD, and YgfK. (a) Structural comparisons of D-aminoacylase (PDB: 3GIQ) and *Cd*UacY. (b) Structural comparisons of *Rhodobacter capsulatus* XDH (PDB: 1JRO) and *Ec*XdhD-YgfM. (c) Structural comparisons of *Ec*YgfK and *Sus*PydA.


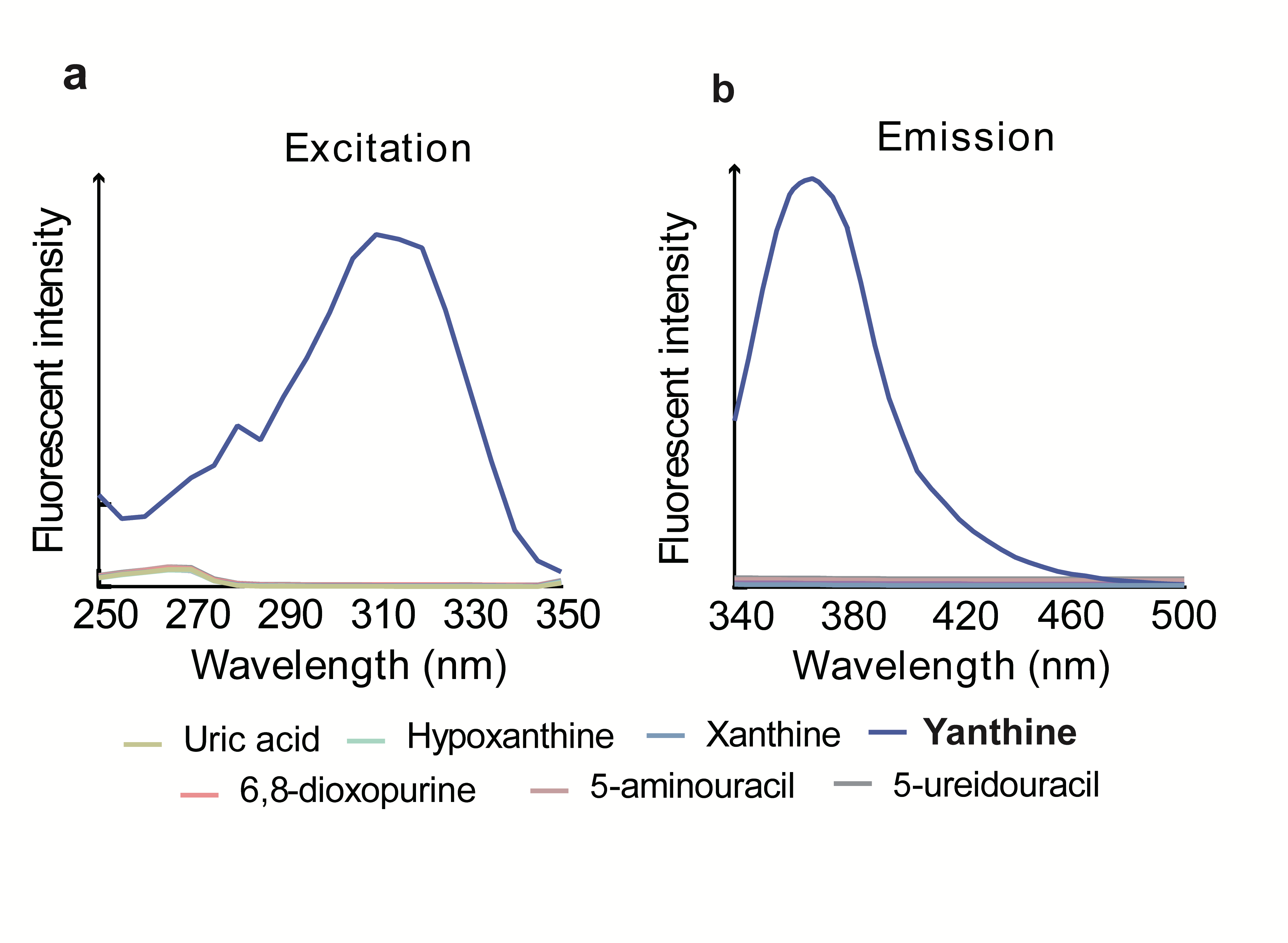


**Supplementary Figure S3** Fluorescence spectrum of purine and pyrimidine derivatives. (a) Excitation spectrum. (b) Emission spectrum.


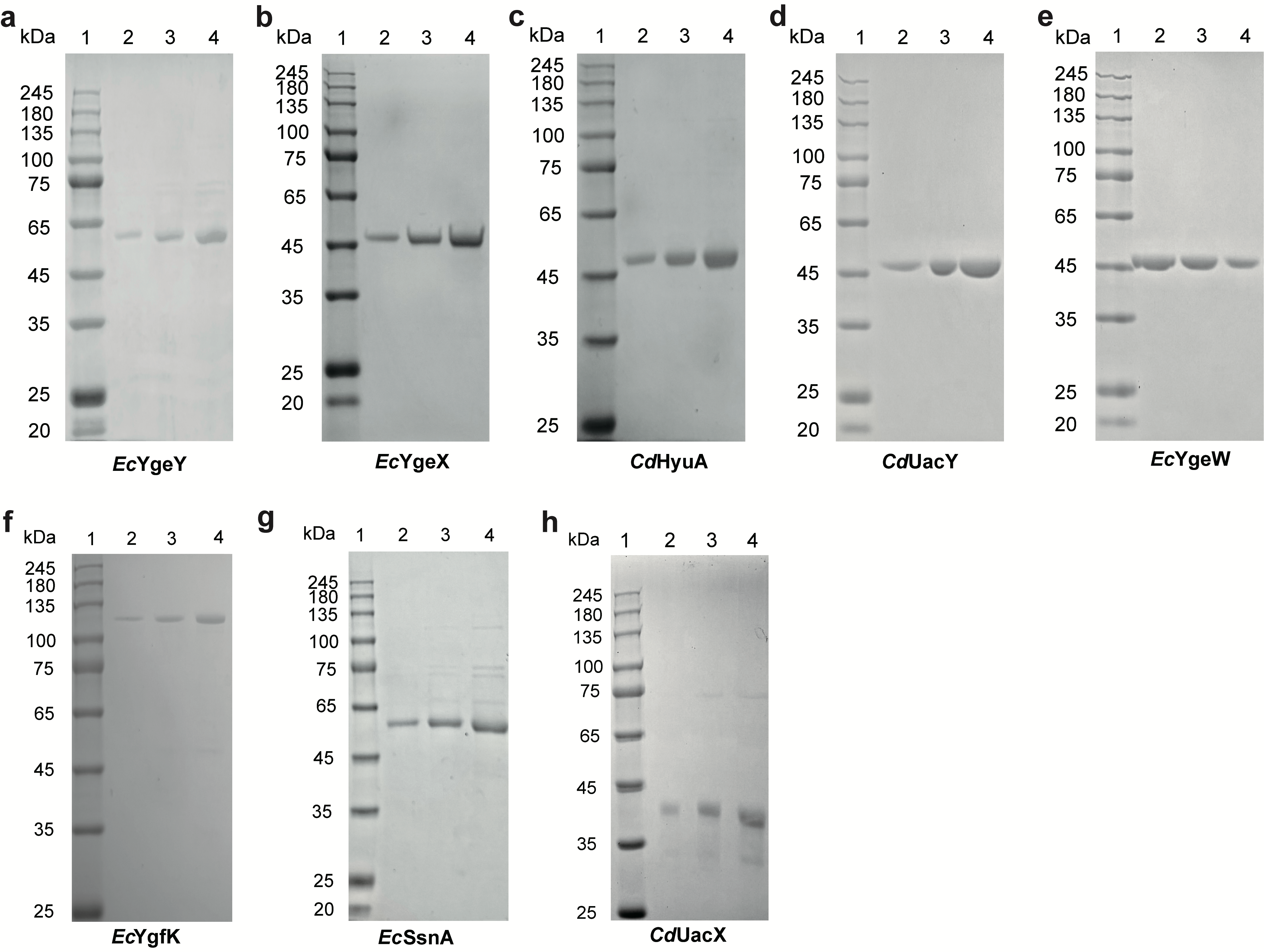


**Supplementary Figure S4** SDS-PAGE analyses of purified proteins used in enzymatic assays and biochemical characterization. (a) *Ec*YgeY. (b) *Ec*YgeX. (c) *Cd*HyuA. (d) *Cd*UacY. (e) *Ec*YgeW. (f) *Ec*YgfK. (g) *Ec*SsnA. (h) *Cd*UacX. Each 4%−20% gradient gel (Bis-Tris) contains protein molecular weight marker and 1, 2, 4 μg of the recombinant protein from lane 1 to lane 4 in order.


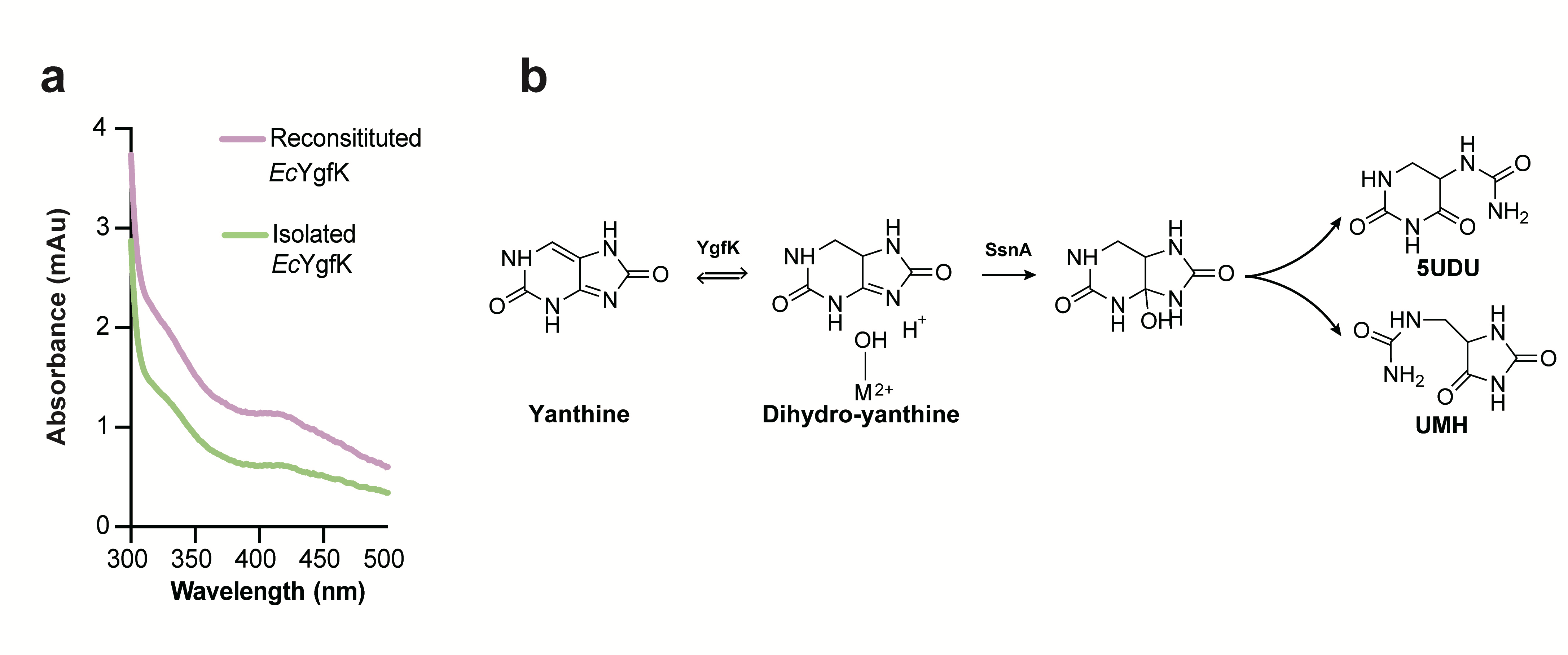


**Supplementary Figure S5** YgfK iron-sulfur cluster and SsnA mechanism. (a) UV-Vis absorption spectra of isolated and reconstituted YgfK. The feature at 410 nm corresponds to [4Fe-4S]^2+^ clusters in reconstituted YgfK. (b) Proposed mechanism for SsnA.


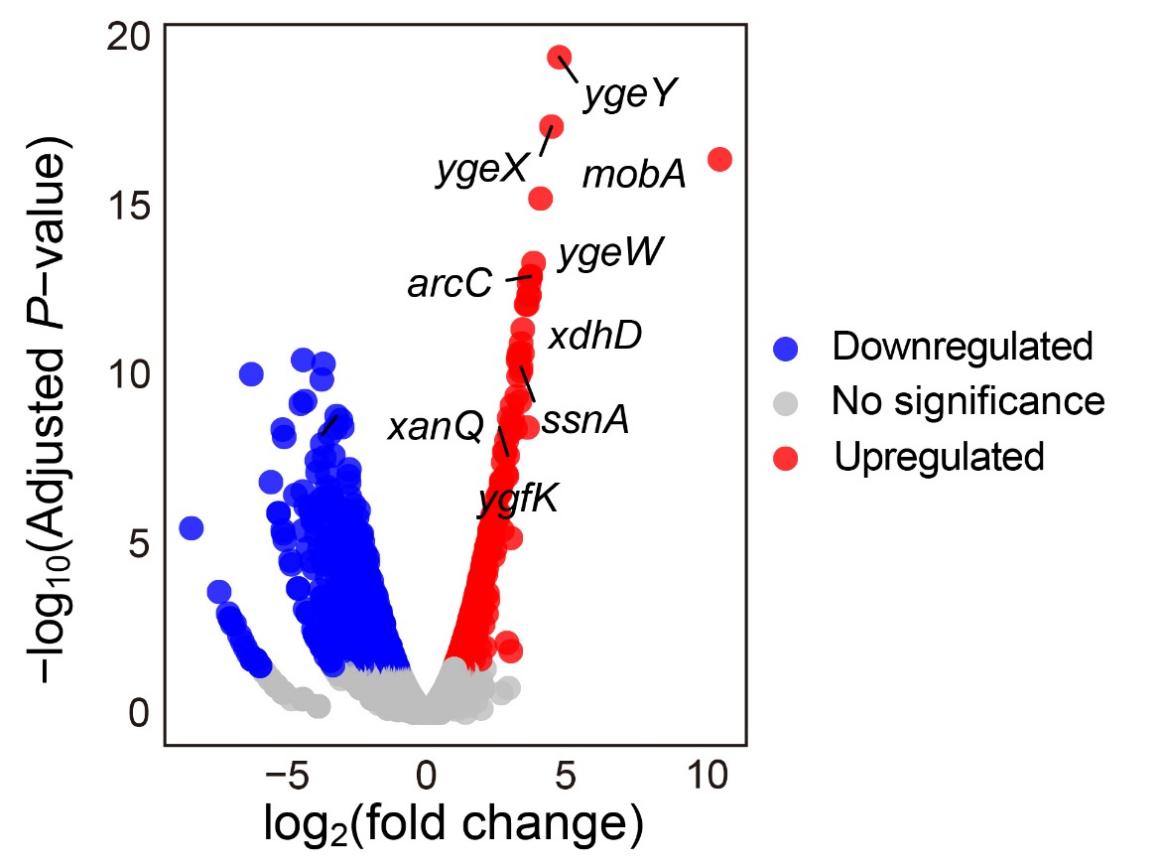


**Supplementary Figure S6** Volcano plot showing differential expression results of uric acid degradation pathway genes in wide-type *EcN* (WT) and CarBT4gout 2.0 strains. RNA-seq results compare responder to non-responder biopsies via DESeq2. Differential expression analysis was performed using DESeq2, applying a two-sided Wald test. *P*-values were adjusted for multiple comparisons using the Benjamini–Hochberg method to control the false discovery rate (FDR). Data are shown as log_2_(fold change) with associated adjusted *P*-values.


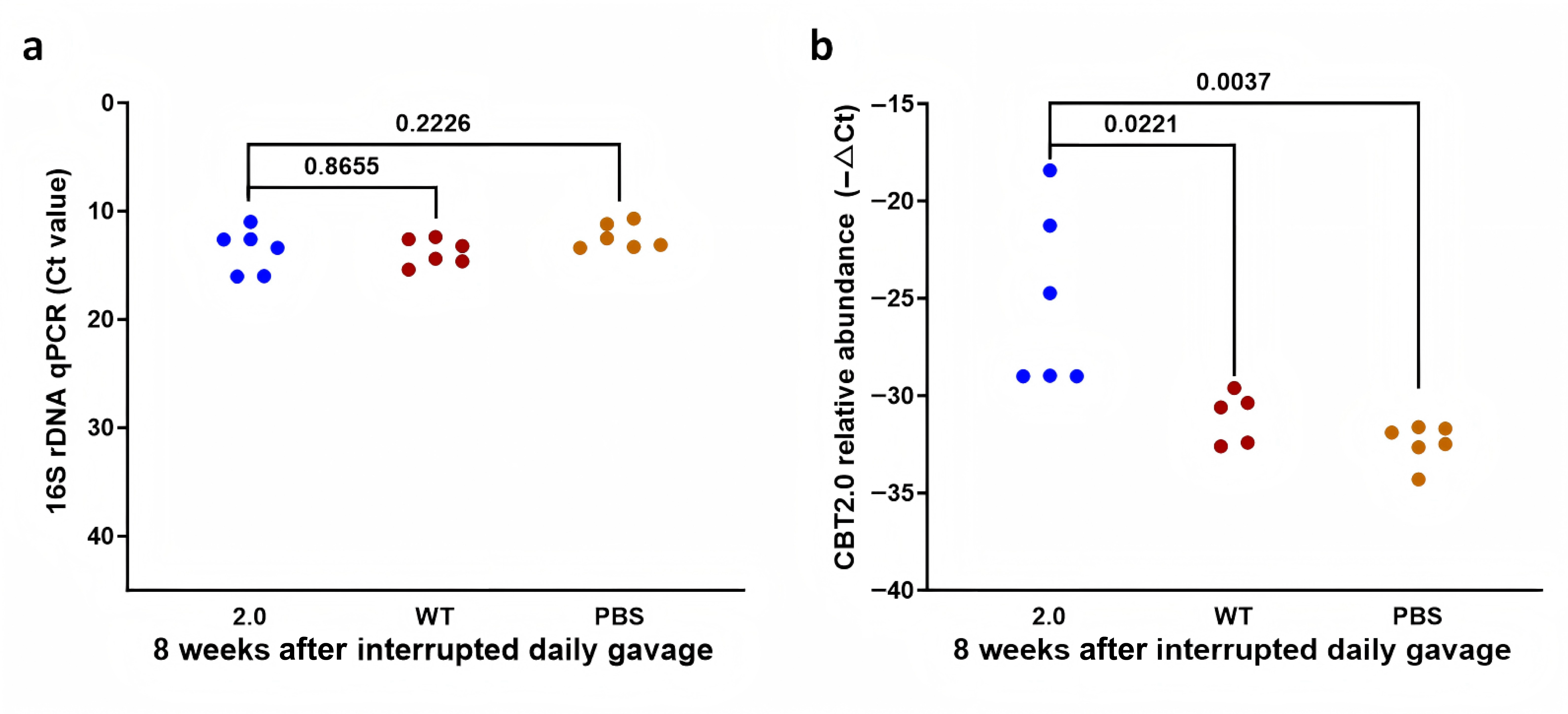


**Supplementary Figure S7** Mouse colonal content bacterial DNA qPCR analysis. (a) Ct values obtained from primers targeting the 16S rDNA. (b) Relative abundance of CBT2.0 in colonal content. Y-axis represents −ΔCt values obtained from −(Ct (*A. indistinctus*) − Ct (16S rDNA genes)), and 45 was used when the Ct value was greater than 45. Each data point corresponds to an individual mouse (*n* =5−6 mice per group) and represents the mean of three technical replicates. Statistical comparisons were performed with two-tailed unpaired *t*-tests, and exact *P*-values are indicated above the brackets. See also Supplementary Table S1.


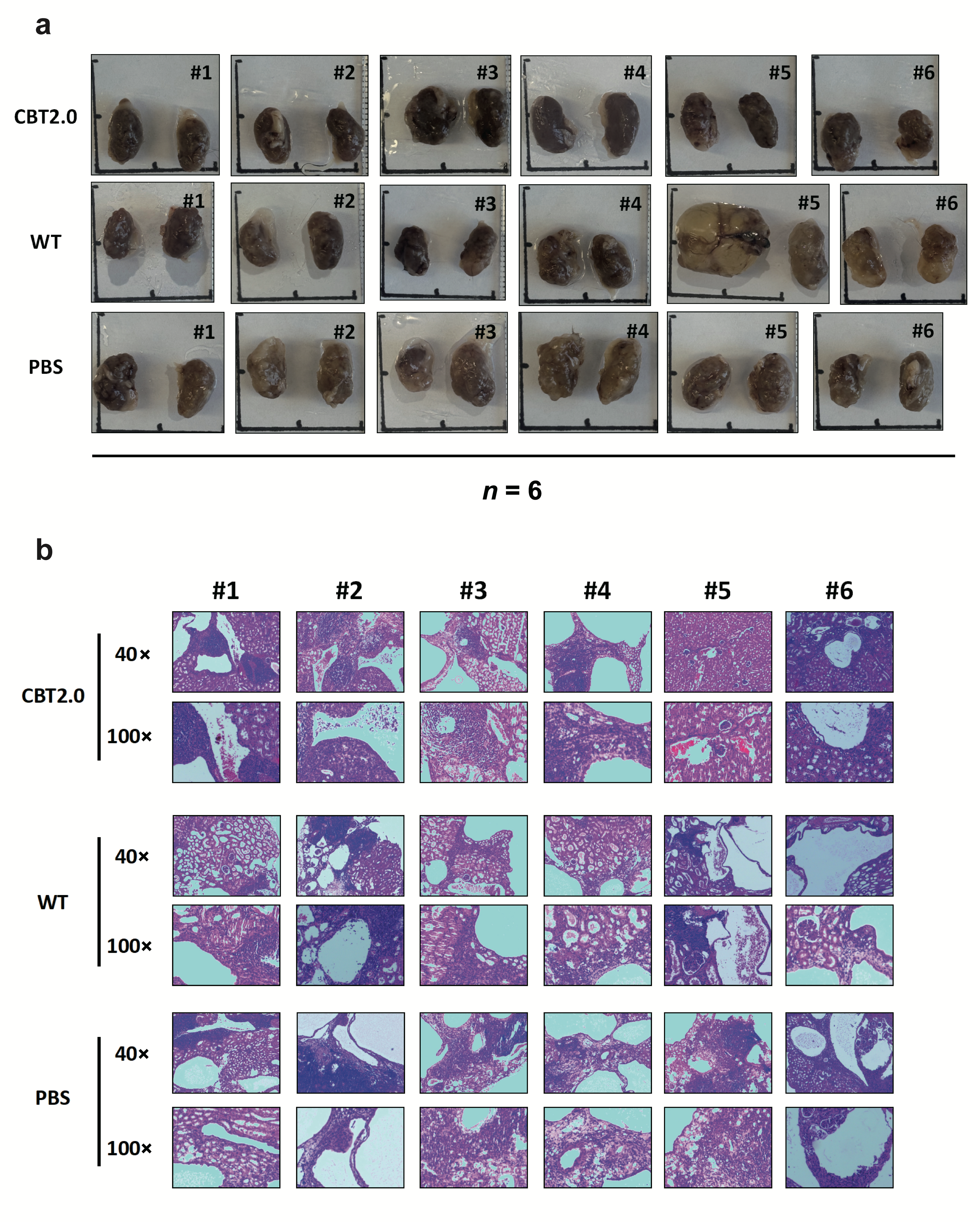


**Supplementary Figure S8** Mouse kidney morphology and histology analysis. (a) Gross appearance of kidneys collected from mice administered with CBT2.0, WT, or PBS (top to bottom, respectively, *n* = 6). Scale bar = 2 cm. (b) Representative hematoxylin and eosin (H&E) staining of kidney tissues from mice in CBT2.0, WT, or PBS groups. Upper pannels: 40× magnification; lower panels: 100× magnification. Quantitative pathology scores are provided in Supplementary Table S1.

**References**

1. Li Q, Sun B, Chen J *et al*. A modified pCas/pTargetF system for CRISPR-Cas9-assisted genome editing in *Escherichia coli*. *Acta Biochim Biophys Sin (Shanghai)* 2021;**53**:620–7.
